# Why Hide AI Use? Psychological Configurations and Explainable Machine Learning Evidence from Marketing Work

**DOI:** 10.3390/bs16060994

**Published:** 2026-06-15

**Authors:** Filiz Mizrak, Turhan Karakaya

**Affiliations:** 1 Management Information Systems, Atlas University, Istanbul 34403, Turkey; 2 Department of Business Administration, Dogus University, Istanbul 34775, Turkey

**Keywords:** AI disclosure silence, workplace AI, employee silence, psychological safety, fuzzy-set Qualitative Comparative Analysis (fsQCA), explainable machine learning

## Abstract

Artificial intelligence (AI) is increasingly embedded in marketing work, yet employees who use AI tools may not always disclose AI’s role in producing their outputs. This study examines AI disclosure silence, defined as employees’ intentional withholding of information about the use, role, or contribution of AI tools in work-related outputs after AI has already been used. Unlike AI avoidance or resistance, this construct concerns post-adoption concealment; unlike general employee silence, it focuses on the hidden technological contribution behind visible work. Drawing on Conservation of Resources Theory and Psychological Safety Theory, the study investigates how threat-based conditions, safety and governance conditions, and AI-related capability are associated with AI disclosure silence. Data were collected through a two-wave survey of 635 marketing employees who actively used AI tools at work. The analysis combined measurement validation, Necessary Condition Analysis (NCA), fuzzy-set Qualitative Comparative Analysis (fsQCA), and explainable machine learning. The findings show that no single condition operated as a strong necessary bottleneck. Instead, AI disclosure silence appeared through multiple pathways involving AI anxiety, fear of negative evaluation, perceived creativity threat, perceived job insecurity, low trust in management, weak psychological safety, and unclear AI policy. SHapley Additive exPlanations (SHAP)-based interpretation further indicated that fear of negative evaluation, AI anxiety, perceived creativity threat, and trust in management had the strongest model-based predictive relevance. The study contributes to workplace AI and employee silence research by positioning AI disclosure silence as an emerging post-adoption disclosure construct. It also highlights the need for clear AI disclosure norms, non-punitive managerial responses, AI-assisted authorship guidelines, and psychologically safe AI-governance practices. The findings should be interpreted as configurational and predictive evidence rather than causal effects, and further scale validation across sectors and cultures is encouraged.

## 1. Introduction

Artificial intelligence (AI) has become part of everyday work. Employees increasingly use AI tools to draft text, summarize information, generate ideas, analyze data, prepare presentations, design visuals, and support decision-making. This shift is especially visible in marketing work, where AI can assist with campaign ideation, advertising copy, social media content, market research, customer analysis, search engine optimization, email communication, and visual design. Yet the growing use of AI does not necessarily mean that employees openly disclose how AI contributes to their work. In many organizations, employees may use AI tools privately while avoiding explicit mention of AI’s role in the final output. This creates a timely workplace psychology issue: AI-supported work may be common, but the actual role of AI may remain hidden.

This study introduces the concept of AI disclosure silence, defined as employees’ intentional withholding of information about the use, role, or contribution of AI tools in producing work-related outputs, despite having used such tools in the work process. The construct is related to employee silence, but it is not identical to it. General employee silence usually refers to withholding opinions, concerns, suggestions, or feedback that may be relevant to organizational learning and change (Morrison & Milliken, 2000; Van Dyne et al., 2003). AI disclosure silence is more specific. It concerns silence about the technological process, authorship, or AI-supported contribution behind a visible work output. It also differs from AI avoidance and AI resistance. Employees who avoid or resist AI may refuse, minimize, or oppose AI use, whereas employees showing AI disclosure silence may actively use AI but choose not to disclose that use. Therefore, the central issue is not AI non-use, but the hidden character of AI-assisted work after adoption has already occurred.

This distinction is theoretically important because AI disclosure silence reflects a new tension in knowledge work: employees may benefit from AI while also fearing that disclosure will weaken the perceived value of their own expertise. In marketing, this tension is particularly salient because employees are often evaluated through creativity, originality, strategic contribution, and professional judgment. If a marketing employee discloses that AI helped develop a campaign idea, advertising text, customer insight, or visual concept, others may question whether the work is genuinely creative, whether the employee is sufficiently competent, or whether the output should be attributed to the employee, the AI tool, or both. Thus, AI disclosure silence is not only a communication issue. It is also connected to technological authorship, professional identity, authenticity anxiety, legitimacy management, and the perceived devaluation of human creativity.

Recent workplace AI research also suggests that employee responses to digital-intelligence transformation cannot be understood only through adoption or usage. Employees’ intentions, identity-related interpretations, and behavioral adjustments are increasingly central to understanding how AI changes work (Wu et al., 2025a). Similarly, studies on human–robot and human–AI collaboration show that intelligent technologies may reshape employees’ perceptions of job security, role expectations, work identity, and behavioral adaptation (Wu et al., 2025b; Xu et al., 2025). These developments make it necessary to examine not only whether employees use AI, but also whether they feel safe and legitimate enough to disclose AI’s involvement in their work.

The psychological foundations of AI disclosure silence can be understood through two complementary theoretical lenses: Conservation of Resources Theory and Psychological Safety Theory. Conservation of Resources Theory suggests that individuals seek to protect valued resources such as professional reputation, self-esteem, job security, autonomy, emotional energy, and social acceptance (Hobfoll, 1989). From this perspective, employees may remain silent about AI use when disclosure is perceived as a possible resource-loss event. For example, disclosure may create fear that one’s creativity, effort, competence, or professional distinctiveness will be questioned. Psychological Safety Theory further explains why disclosure depends on the interpersonal climate of the workplace. Employees are more likely to speak openly when they believe that doing so will not result in embarrassment, punishment, or negative judgment (Edmondson, 1999). In contrast, when managerial trust is low, AI-use rules are unclear, or disclosure norms are ambiguous, silence may appear to be the safest response.

Marketing is used in this study as a theoretically rich case of knowledge work rather than as a claim that AI disclosure concerns are unique only to marketing. Similar tensions may also arise among designers, consultants, researchers, teachers, programmers, and other knowledge workers. However, marketing provides a suitable context because AI-supported work often concerns visible, evaluative, and creativity-oriented outputs. Marketing employees are expected to be fast, original, persuasive, and strategically insightful, while also adapting to rapidly evolving AI tools. This makes the marketing context especially useful for examining how AI use intersects with creativity threat, fear of negative evaluation, job insecurity, trust, policy clarity, psychological safety, and AI self-efficacy.

Despite the relevance of this issue, hidden AI use remains underdeveloped as a workplace psychology problem. Existing research has largely focused on AI adoption, AI acceptance, AI anxiety, trust in AI, technostress, or resistance to automation. These perspectives are valuable, but they do not fully explain what happens after employees begin using AI and then decide whether to disclose or conceal AI’s contribution. This study addresses this gap by examining AI disclosure silence as an emerging construct. The study does not claim that the construct is fully settled; rather, it provides an initial empirical investigation and recognizes the need for further validation across sectors, cultures, and organizational AI-governance contexts.

The study also responds to the possibility that AI disclosure silence may not follow one simple linear pathway. Employees may remain silent for different reasons. Some may conceal AI use because of fear of negative evaluation and perceived creativity threat. Others may do so because of job insecurity, low trust in management, low psychological safety, unclear AI policies, or limited AI self-efficacy. For this reason, the study organizes the explanatory conditions into three theoretical clusters: threat-based conditions, safety and governance conditions, and capability-related conditions. This clustering helps avoid treating all variables as disconnected predictors and allows the framework to reflect the psychological complexity of AI disclosure decisions.

Methodologically, the study uses a two-wave survey design among marketing employees who actively use AI tools at work. The two-wave structure provides temporal separation between the psychological and organizational conditions and the outcome measure, although it does not eliminate all limitations associated with self-reported data. The analytical strategy is sequential rather than fragmented. Measurement validity is first assessed; then Necessary Condition Analysis (NCA) examines whether any condition functions as a necessary bottleneck; fuzzy-set Qualitative Comparative Analysis (fsQCA) identifies alternative configurations associated with high and low AI disclosure silence; and explainable machine-learning analysis evaluates model-based predictive relevance. SHapley Additive exPlanations (SHAP) analysis is used to interpret how each condition contributes to the model’s predictions, not to make causal claims about employee behavior.

This study makes three main contributions. First, it introduces AI disclosure silence as an emerging workplace AI psychology construct and clarifies its boundaries in relation to defensive silence, knowledge hiding, AI avoidance, and AI resistance. Second, it extends the discussion of workplace AI from adoption and acceptance to post-adoption disclosure, showing that employees may use AI while remaining reluctant to reveal its role in their work. Third, it offers a configurational and predictive perspective on AI disclosure silence by examining how threat perceptions, safety and governance conditions, and AI-related capability combine in different ways. Practically, the study highlights the need for organizations to develop AI transparency cultures, non-punitive disclosure norms, clear AI authorship expectations, and psychologically safe governance practices for AI-supported work.

## 2. Literature Review and Theoretical Background

AI has moved from a specialized technological resource to a routine element of everyday work behavior. In marketing, this transformation is visible in campaign ideation, advertising copy, social media content, customer analysis, market research, search engine optimization (SEO) writing, email communication, visual design, and presentation preparation. Yet the growing use of AI does not mean that employees disclose its role openly. In many organizations, AI use is encouraged informally but governed ambiguously, which creates a disclosure dilemma: employees may rely on AI privately while leaving its contribution invisible.

Marketing offers a useful context for examining this dilemma because marketing work is strongly associated with creativity, originality, persuasion, strategic thinking, and professional expertise. When AI contributes to a slogan, campaign idea, visual concept, or customer insight, employees may worry that disclosure will reduce the perceived value of their own contribution. The central concern is therefore not only whether employees adopt AI, but whether they feel safe, legitimate, and confident enough to acknowledge AI-supported work practices.

### 2.1. AI Disclosure Silence as an Emerging Construct

AI disclosure silence refers to employees’ intentional withholding of information about the use, role, or contribution of AI tools in producing work-related outputs after AI has already been used. The construct builds on the employee silence literature, where silence is understood as withholding relevant ideas, concerns, opinions, or information that could affect organizational learning and change (Morrison & Milliken, 2000; Van Dyne et al., 2003). However, AI disclosure silence is narrower and more specific. It concerns the concealed technological process behind visible work outputs rather than the withholding of general opinions or suggestions.

This distinction is important because AI disclosure silence is closest to defensive silence but not identical to it. Defensive silence involves self-protective withholding to avoid negative consequences; AI disclosure silence shares this protective logic but focuses on AI-supported authorship, contribution, and production. Recent reviews and meta-analytic work show that employee silence has multiple antecedents and outcomes and remains strongly connected to voice, well-being, burnout, and organizational functioning (Dehkharghani et al., 2023; Hao et al., 2022; Lainidi et al., 2023; Lainidi et al., 2025). These insights justify extending silence theory to AI-supported work, where concealment may arise from uncertainty about originality, ethics, and professional evaluation.

AI disclosure silence also differs from knowledge hiding, AI avoidance, and AI resistance. Knowledge hiding concerns withholding requested knowledge from others, whereas AI disclosure silence concerns concealing AI’s role in one’s own completed work. AI avoidance and AI resistance describe non-use, rejection, or opposition to AI; AI disclosure silence occurs after adoption, when employees use AI but avoid revealing its contribution. Recent workplace AI studies similarly show that employee responses to intelligent technologies involve intentions, identity, behavioral adaptation, and organizational support rather than simple adoption decisions (Wu et al., 2025a; Wu et al., 2025b; Xu et al., 2025; Nguyen et al., 2026). Table 1 distinguishes AI disclosure silence from related constructs and clarifies its conceptual scope.

### 2.2. Resource Protection and Psychological Safety

The first theoretical foundation is the Conservation of Resources Theory. This theory argues that individuals seek to obtain, retain, and protect valued resources, and that stress emerges when these resources are threatened or lost (Hobfoll, 1989). In AI-supported marketing work, disclosure can be perceived as a possible resource-loss event because it may threaten creative identity, competence image, job security, reputation, autonomy, emotional energy, and social acceptance. From this view, silence is not necessarily irrational; it can be interpreted as a protective response to perceived professional vulnerability.

Recent studies using resource-based reasoning reinforce this interpretation. Work on job demands, well-being, burnout, psychological capital, social support, and work engagement suggests that employees evaluate workplace change through possible gains and losses in valued resources (Demerouti, 2025; G. Chen et al., 2024; Nemțeanu et al., 2022). AI can therefore function as both a resource and a demand. It may improve speed, idea generation, and productivity, while also increasing anxiety about replacement, originality, or loss of professional control.

The second theoretical foundation is Psychological Safety Theory. Psychological safety refers to a shared belief that the work environment is safe for interpersonal risk-taking (Edmondson, 1999). Disclosing AI use is such a risk because employees cannot know whether managers and colleagues will interpret AI assistance as responsible innovation, efficient work practice, overreliance on technology, or reduced effort. When psychological safety is weak, silence may become a safer strategy than transparency.

This logic is supported by research linking psychological safety with trust, job satisfaction, inclusive leadership, innovation, and thriving at work (Mitterer & Mitterer, 2023; T. Li & Tang, 2022; Zhan et al., 2025). It is also relevant to AI contexts, where psychologically unsafe implementation may intensify negative employee outcomes. For example, recent work connects AI adoption with employee depression through psychological safety and ethical leadership (Kim et al., 2025). These findings suggest that AI disclosure is shaped not only by individual attitudes toward technology, but also by whether the surrounding climate allows employees to discuss AI-related experimentation, uncertainty, and mistakes.

### 2.3. Psychological and Organizational Conditions

The explanatory conditions are organized into three clusters: threat-based conditions, safety and governance conditions, and capability-related conditions. This clustered structure avoids treating the framework as a list of unrelated variables and responds to the possibility that AI disclosure silence reflects overlapping forms of threat, safety, and capability.

The first threat-based condition is AI anxiety. AI anxiety captures discomfort, uncertainty, and concern about AI’s implications for learning, replacement, and work identity (Wang & Wang, 2022). In marketing, AI anxiety may be intensified because AI increasingly supports tasks that were traditionally associated with human creativity and strategic judgment. Recent studies on AI technostress, AI-related job insecurity, AI-use anxiety, emotional exhaustion, and employee well-being similarly show that AI can become a psychological demand rather than only a productivity tool (Huvanandana & Charoensukmongkol, 2026; Lyu et al., 2026; Ladhari et al., 2026).

Fear of negative evaluation is another threat-based condition. It reflects concern that others will judge the employee unfavorably (Leary, 1983). In marketing, this concern can be strong because outputs are visible, subjective, and often judged by managers, clients, and audiences. This fear also intersects with creativity-related identity. Creative self-efficacy research shows that confidence in creative ability is an important psychological resource (Tierney & Farmer, 2002). Employees may therefore conceal AI use when they believe disclosure will make an idea, campaign, or text appear less original or less authentically theirs.

Perceived job insecurity represents another disclosure threat. Job insecurity reflects concern about the stability of one’s employment and future work role (Vander Elst et al., 2014). In AI-supported marketing, insecurity may involve fear of job loss, task loss, career stagnation, reduced bargaining power, or declining professional relevance. Recent research also shows that job security shapes workplace safety perception, engagement, and autonomy (Bhatti, 2024). Employees who feel replaceable may avoid disclosing AI use because disclosure could appear to provide evidence that parts of their work can be automated.

The safety and governance cluster includes trust in management, psychological safety, and AI policy clarity. Trust in management refers to the willingness to be vulnerable to managerial action based on perceived ability, benevolence, and integrity (Mayer et al., 1995). AI disclosure requires this vulnerability because managers may use disclosed AI use constructively or punitively. Recent studies on workplace generative AI, AI-driven leadership, and human–AI interaction show that explanations, ethics, self-efficacy, and algorithmic leadership shape trust and disengagement in AI-related work contexts (Gibbard et al., 2026; Khassawneh et al., 2026; C. S. Lin et al., 2025).

AI policy clarity reflects whether employees understand what AI tools may be used, when AI use should be disclosed, how AI-generated content should be checked, and who is accountable for errors. This condition extends the classic role ambiguity literature, which shows that unclear expectations create strain in complex organizations (Rizzo et al., 1970). Recent work on AI-driven digital monitoring, organizational listening, and public workplace monitoring further emphasizes that employees interpret AI-related organizational signals when deciding how to behave (Y. Li et al., 2026; Dong et al., 2024; Boykin et al., 2026). When guidance is unclear, employees may develop informal rules that include hidden or silent use.

The capability-related condition is AI/digital self-efficacy. Computer self-efficacy refers to beliefs about one’s ability to use computer technologies successfully (Compeau & Higgins, 1995). In the AI context, this includes confidence in prompting, evaluating AI outputs, detecting inaccuracies, protecting confidential data, and integrating AI suggestions with human judgment. Recent studies show that AI usage can reshape self-efficacy and risk-taking, while AI literacy and digital literacy influence intentions, identity, participation, and trust through self-efficacy mechanisms (Han et al., 2025; Duong, 2025; X. Bai & Yang, 2025). Employees with stronger AI self-efficacy may be better able to explain how they used AI and why the final output remains professionally valid.

### 2.4. Ethical and Governance Implications of Hidden AI Use

AI disclosure silence should not automatically be treated as unethical AI use. Employees may conceal AI involvement because disclosure expectations are unclear, because they fear reputational loss, or because they do not trust how managers will interpret AI-supported work. At the same time, hidden AI use can create organizational risks. Managers may be unable to evaluate whether AI-generated content is accurate, original, ethical, legally safe, brand-consistent, or appropriately reviewed.

Recent studies highlight the ethical complexity of workplace AI use. Ethical perceptions of generative AI can shape work outcomes through moral rumination and AI-supported autonomy, while workplace AI use may promote unethical outcomes under some conditions (J. Y. Bai et al., 2025; Zhao et al., 2026). Research on AI-use dilemmas and deviant workplace behavior also shows that AI use may have unintended behavioral consequences (Chi et al., 2026). In addition, digital silence may prevent organizations from detecting structural bias, misuse, or harm in AI-mediated systems (Walle, 2025). These studies reinforce the argument that AI disclosure silence has governance consequences as well as psychological roots.

AI should nevertheless not be framed only as a threat. Recent research shows that AI awareness, AI identity, knowledge management, and workplace AI use can support innovative behavior, proactive behavior, and citizenship outcomes, although these effects can be double-edged (Yang et al., 2026; Karkoulian & Boulos, 2026; Q. Lin & He, 2026; Qin et al., 2025; Zhang et al., 2025). Practice-oriented and interpretive studies also suggest that employees are increasingly dependent on AI while organizations struggle to develop shared norms for AI-supported work (Bilderback, 2025; Kodagoda, 2026; Gabelaia et al., 2024). This gray zone is precisely where AI disclosure silence can emerge.

### 2.5. Configurational and Predictive Logic

AI disclosure silence is unlikely to follow one single pathway. One employee may hide AI use because creativity threat and fear of negative evaluation are high. Another may remain silent because job insecurity is high and managerial trust is low. Another may conceal AI use because policy clarity is weak or because AI self-efficacy is low. These alternative routes reflect equifinality, meaning that different combinations of conditions can lead to the same outcome.

The analytical logic follows this assumption. Exploratory and confirmatory factor analyses first assess the measurement structure and construct distinctiveness, with conventional fit-index guidance used to evaluate model fit (Hu & Bentler, 1999). Necessary Condition Analysis then examines whether any condition functions as a necessary but not sufficient bottleneck for AI disclosure silence (Dul, 2016, 2021; Richter et al., 2020). fsQCA examines sufficient configurations and is appropriate because it allows conditions to combine differently across cases rather than assuming one uniform net effect (Ragin, 2008; Fiss, 2011; Pappas & Woodside, 2021).

The predictive stage adds a complementary layer. Random Forest and extreme gradient boosting (XGBoost) are useful for detecting complex, non-linear prediction patterns (Breiman, 2001; T. Chen & Guestrin, 2016). SHAP then supports interpretation by estimating how much each predictor contributes to the model’s predictions (Lundberg & Lee, 2017). Because AI disclosure silence is a newly operationalized construct, the study also treats measurement validation cautiously and recognizes the need for further psychometric testing, consistent with recent AI-related scale-development work (Ayasrah et al., 2026).

Taken together, the literature positions AI disclosure silence as a psychologically motivated form of silence in AI-supported marketing work. Conservation of Resources Theory explains why employees may conceal AI use to protect valued resources, Psychological Safety Theory explains why disclosure depends on interpersonal safety, and recent workplace AI research shows that AI use is embedded in trust, identity, governance, ethics, and capability. The framework therefore integrates threat-based, safety/governance, and capability-related conditions as interrelated pathways rather than isolated predictors.

## 3. Conceptual Framework and Research Questions

### 3.1. Conceptual Logic

This study frames AI disclosure silence as a protective post-adoption disclosure behavior. Employees may use AI tools in marketing work but avoid revealing AI’s role in their outputs because disclosure may expose them to judgment, misunderstanding, or professional harm. This behavior does not necessarily indicate AI resistance, unethical conduct, or lack of competence. Rather, it may reflect uncertainty about disclosure norms and concern over how AI-supported work will be interpreted by managers, colleagues, or clients.

The conceptual logic is grounded in two complementary theories. Conservation of Resources Theory suggests that employees seek to protect valued resources such as professional reputation, creative identity, job security, autonomy, social acceptance, and perceived competence (Hobfoll, 1989). From this perspective, disclosing AI use may be perceived as risky when employees believe that AI involvement could make their work appear less original, less effortful, or more replaceable. Psychological Safety Theory adds the interpersonal dimension of disclosure. Employees are more likely to discuss AI-supported work when they believe that the workplace is safe for admitting uncertainty, explaining new work practices, and taking interpersonal risks without fear of embarrassment or punishment (Edmondson, 1999).

The framework organizes the conditions associated with AI disclosure silence into three clusters. The first cluster includes threat-based conditions: AI anxiety, fear of negative evaluation, perceived creativity threat, and perceived job insecurity. These conditions reflect different ways in which AI disclosure may be interpreted as a risk to status, identity, employability, or professional value. The second cluster includes safety and governance conditions: psychological safety, trust in management, and AI policy clarity. These conditions reflect whether disclosure is perceived as acceptable, fair, and non-punitive. The third cluster is capability-related and is represented by AI/digital self-efficacy, which reflects whether employees feel capable of using AI responsibly and explaining their AI-supported work.

The study assumes that AI disclosure silence may arise through different combinations of these conditions rather than through one single pathway. For instance, some employees may remain silent mainly because of creativity threat and fear of negative evaluation, while others may do so because of job insecurity, low trust, unclear AI policies, or limited AI self-efficacy. This configurational logic reflects equifinality, where different psychological and organizational pathways may be associated with the same outcome. It also allows the study to examine whether the conditions associated with high AI disclosure silence differ from those associated with low AI disclosure silence.

The analytical strategy follows this conceptual logic. Necessary Condition Analysis is used to examine whether any condition functions as a bottleneck for AI disclosure silence. fsQCA is used to identify sufficient combinations of conditions associated with high and low AI disclosure silence. Random Forest, XGBoost, and SHAP are used to assess model-based predictive relevance and to interpret how each condition contributes to the machine-learning model’s predictions. These methods are used sequentially to connect necessity, configurational sufficiency, and predictive interpretation, rather than as separate or disconnected techniques.

### 3.2. Research Questions

The study addresses four research questions:

Research question 1 (RQ1): Which psychological and organizational conditions are necessary for high AI disclosure silence among marketing employees who use AI tools at work?

This question examines whether any condition functions as a necessary bottleneck. For example, it considers whether high fear of negative evaluation, high perceived creativity threat, high AI anxiety, high job insecurity, low psychological safety, unclear AI policy, low trust in management, or low AI self-efficacy must be present for high AI disclosure silence to occur.

RQ2: Which combinations of threat-based, safety/governance, and capability-related conditions are sufficient for high AI disclosure silence in marketing work?

This question reflects the configurational nature of the framework. AI disclosure silence may not be associated with one condition alone; instead, it may emerge through different psychological profiles, such as an identity-protection pathway, an insecurity-driven pathway, or a low-trust concealment pathway.

RQ3: Which conditions have the strongest model-based predictive relevance for AI disclosure silence among marketing employees?

This question focuses on predictive interpretation. Using machine-learning models and SHAP analysis, the study examines how each condition contributes to the model’s predictions of AI disclosure silence. The results are interpreted as predictive evidence, not as causal effects.

RQ4: Are the configurations associated with high AI disclosure silence different from those associated with low AI disclosure silence?

This question examines configurational asymmetry. Low AI disclosure silence may not simply be the opposite of high AI disclosure silence. Openness may require a different combination of conditions, such as high psychological safety, clear AI policy, high trust in management, and stronger AI self-efficacy.

## 4. Methodology

### 4.1. Research Design and Unit of Analysis

This study uses a quantitative two-wave survey design to examine the psychological and organizational conditions associated with AI disclosure silence among marketing employees. A survey design is suitable because the study focuses on employees’ perceptions, emotions, and workplace experiences, including AI anxiety, fear of negative evaluation, perceived creativity threat, perceived job insecurity, psychological safety, trust in management, AI policy clarity, and AI/digital self-efficacy. These constructs reflect how employees interpret AI-supported work in their own organizational settings and cannot be directly observed through secondary data or organizational records.

The unit of analysis is the individual employee. Organizational conditions such as psychological safety, trust in management, and AI policy clarity are measured through employees’ perceptions rather than as objective organization-level characteristics. This distinction is important because the study does not claim to measure organizational AI governance directly; instead, it examines how employees perceive the social and managerial conditions surrounding AI use.

The two-wave structure was used to create temporal separation between the explanatory conditions and the outcome variable. In Wave 1, participants reported the psychological and organizational conditions of the study. In Wave 2, the same participants reported their level of AI disclosure silence. This design reduces the risk of measuring all constructs at the same time and helps limit some forms of common method bias. However, it does not eliminate all limitations associated with self-reported survey data, including same-source bias and social desirability. For this reason, the findings are interpreted as associations and model-based predictive patterns rather than as causal effects.

The study focuses on employees who already use AI-based tools such as ChatGPT (OpenAI, San Francisco, CA, USA), Gemini (Google LLC, Mountain View, CA, USA), Microsoft Copilot (Microsoft Corporation, Redmond, WA, USA), Canva AI (Canva Pty Ltd., Sydney, Australia), Midjourney (Midjourney, Inc., San Francisco, CA, USA), Jasper (Jasper AI, Inc., Rollingwood, TX, USA), Grammarly AI/Superhuman (Superhuman Platform Inc., San Francisco, CA, USA), or similar applications for marketing-related activities. Marketing is treated as a theoretically relevant knowledge-work context because marketing outputs are often evaluated in terms of creativity, originality, persuasion, and professional judgment. AI-supported marketing work may therefore raise concerns about authorship, competence, professional image, and the perceived value of human contribution. At the same time, the study does not assume that AI disclosure silence is limited to marketing. Similar disclosure concerns may also arise in other knowledge-work contexts where AI supports visible and evaluative outputs.

**Figure 1:**
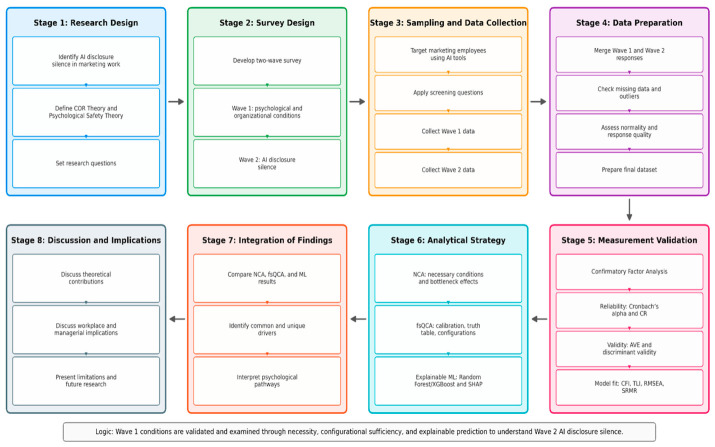
Workflow of the study. Note. AI = artificial intelligence; COR = Conservation of Resources; NCA = Necessary Condition Analysis; fsQCA = fuzzy-set Qualitative Comparative Analysis; ML = machine learning.

The analytical design follows a sequential logic. First, the measurement structure is assessed through exploratory and confirmatory procedures, together with reliability, convergent validity, and discriminant validity checks. Second, Necessary Condition Analysis examines whether any condition functions as a bottleneck for high AI disclosure silence. Third, fsQCA identifies sufficient configurations associated with high and low AI disclosure silence. Finally, explainable machine-learning analysis assesses model-based predictive relevance, and SHAP is used to interpret how each condition contributes to the model’s predictions. These methods are used to answer different research questions in a connected sequence rather than as independent or competing techniques. Figure 1 presents the workflow of the study.

### 4.2. Sample, Recruitment, and Procedure

Data were collected through a two-wave online survey with employees working in marketing-related roles and actively using AI tools in their work tasks. Participants were recruited through professional networks using a non-probability sampling approach. Therefore, the sample should not be interpreted as statistically representative of the full marketing workforce. This limitation is relevant because AI-use norms, disclosure expectations, and managerial responses may differ across organizations, industries, and corporate cultures.

The target group included employees working in advertising agencies, digital marketing agencies, corporate marketing departments, e-commerce companies, media and creative agencies, consulting firms, and other organizations where marketing activities are performed. The first wave was conducted between 3 November and 24 November 2025, and the second wave was conducted between 5 January and 26 January 2026. The six-week interval created temporal separation between the psychological and organizational conditions measured in Wave 1 and AI disclosure silence measured in Wave 2.

Before entering the questionnaire, respondents received an information and informed consent statement explaining the purpose of the research, the voluntary nature of participation, the anonymous handling of responses, and their right to withdraw before submitting the survey. Participation continued only after electronic informed consent was provided. Because the study examined a potentially sensitive workplace behavior—whether employees disclose or hide AI use—anonymity and confidentiality were emphasized to reduce evaluation concerns and social desirability pressure.

Two screening questions were used. First, respondents were asked whether they currently worked in a marketing-related role. Second, they were asked whether they used AI-based tools such as ChatGPT, Gemini, Microsoft Copilot, Canva AI, Midjourney, Jasper, Grammarly AI, or similar applications for marketing-related work tasks. Only respondents who answered “yes” to both questions were eligible. This ensured that the study focused on employees with direct experience of AI-supported marketing work rather than respondents reporting only general attitudes toward AI.

In Wave 1, participants completed the measures for AI anxiety, fear of negative evaluation, perceived creativity threat, perceived job insecurity, psychological safety, AI policy clarity, AI/digital self-efficacy, and trust in management. Demographic and work-related information was also collected in Wave 1. In Wave 2, the same participants completed the AI disclosure silence scale. To match responses across the two waves while preserving anonymity, participants used a self-generated identification code. No personally identifying information was collected.

The initial Wave 1 dataset included 691 eligible respondents. After the Wave 2 follow-up, matching procedure, and data-quality screening, 635 complete matched responses remained in the final dataset. Fifty-six responses were removed because they could not be matched across waves, contained incomplete responses, or failed data-quality checks. This corresponds to an attrition/removal rate of approximately 8.1%. The final sample size was considered adequate for the planned measurement validation, Necessary Condition Analysis, fsQCA, and explainable machine-learning analyses.

Table 2 presents the demographic, professional, organizational, and AI-use profile of the final matched sample. The sample included respondents across gender, age, education level, position level, work experience, marketing experience, organization type, organization size, and AI-use frequency.

Table 3 reports the AI tools and AI-supported marketing tasks selected by participants. Since participants could select more than one AI tool and task, the percentages in Table 3 do not sum to 100.

### 4.3. Measures and Scale Development

All study variables were measured using a seven-point Likert scale ranging from 1 = strongly disagree to 7 = strongly agree. Higher scores indicated higher levels of the relevant construct. In line with the two-wave design, the psychological and organizational conditions were measured in Wave 1, whereas AI disclosure silence was measured in Wave 2. Established scales were used where possible and adapted to the context of AI-supported marketing work.

Because AI disclosure silence is an emerging construct, its measurement required particular care. The scale was developed by adapting the logic of employee silence to the specific context of AI-supported work. The items were worded to capture whether employees intentionally avoid revealing the role of AI in their work-related outputs. Therefore, the scale does not measure AI avoidance, AI resistance, or general unwillingness to use AI. It measures silence about AI use after AI has already been used in the work process. This distinction is important because employees may actively use AI tools while still concealing AI’s contribution to their outputs.

The AI disclosure silence items focused on the withholding of information about AI-supported authorship, process, or contribution. The wording reflected disclosure to managers, colleagues, clients, or the organization more generally. This operationalization follows the self-protective logic of employee silence but applies it to a distinct object of concealment: the role of AI in producing visible work outcomes. Since the construct is still new, the scale is treated as an initial empirical operationalization rather than a fully established instrument. 

Table 4 reports the descriptive statistics of the main constructs, including mean, standard deviation, skewness, and kurtosis values. These results provide an initial view of the distributional properties of the variables before the measurement validation and main analyses. 

Table 5 presents the measurement constructs, wave structure, codes, number of items, adaptation sources, and representative sample items.

The threat-based conditions included AI anxiety, fear of negative evaluation, perceived creativity threat, and perceived job insecurity. AI anxiety captured employees’ discomfort and concern about the consequences of AI for marketing work. Fear of negative evaluation measured concern that managers, colleagues, or clients may judge employees unfavorably if AI use is disclosed. Perceived creativity threat measured whether employees believed that AI use could weaken their image as creative professionals. Perceived job insecurity assessed concern that AI could reduce job stability, career opportunities, or professional value.

The safety and governance conditions included psychological safety, AI policy clarity, and trust in management. Psychological safety measured whether employees felt safe discussing AI use in their team. AI policy clarity measured whether organizational expectations about AI use, disclosure, and checking of AI-generated outputs were clear. Trust in management measured whether employees believed that managers would respond fairly and constructively if AI use were disclosed.

The capability-related condition was AI/digital self-efficacy. This construct measured employees’ confidence in using AI tools effectively, responsibly, and critically in marketing tasks. It was included because employees who feel more capable of using and evaluating AI may also feel more able to explain their AI-supported work.

The study also included demographic, professional, organizational, and AI-use variables as controls: gender, age group, education level, position level, total work experience, marketing experience, organization type, organization size, AI-use frequency, AI tools used, and AI-supported marketing tasks. These variables were included because disclosure behavior may vary depending on professional background, organizational context, and the type or frequency of AI use.

Given the conceptual proximity among several constructs, especially AI anxiety, fear of negative evaluation, perceived creativity threat, perceived job insecurity, psychological safety, and trust in management, the measurement model was examined carefully. Exploratory factor analysis, confirmatory factor analysis, reliability tests, convergent validity, and discriminant validity checks were used to assess whether the constructs were empirically distinguishable. This was particularly important for AI disclosure silence because the construct is newly operationalized and may appear close to defensive silence or other self-protective workplace behaviors.

### 4.4. Data Screening and Common Method Considerations

Before the main analyses, the two-wave dataset was screened to ensure that the responses were complete, consistent, and suitable for measurement validation, Necessary Condition Analysis, fsQCA, and explainable machine-learning analysis. The screening procedure included checks for two-wave matching, missing data, valid response ranges, duplicate codes, outliers, normality, common method bias, and response-pattern problems.

The initial Wave 1 dataset included 691 eligible respondents who met the screening criteria of working in a marketing-related role and using AI tools for marketing-related tasks. After the Wave 2 follow-up and matching procedure, 635 complete matched responses remained in the final dataset. Fifty-six responses were removed because they could not be matched across waves, contained incomplete responses, or failed data-quality checks. This corresponds to an attrition/removal rate of approximately 8.1%. The final dataset contained no missing values in the main construct items, and all Likert-scale responses were within the valid response range of 1 to 7.

Outliers were examined at both univariate and multivariate levels. For univariate outliers, standardized scores were calculated for the composite construct means. No construct-level case exceeded the ±3.29 threshold. Multivariate outliers were assessed using Mahalanobis distance across the main constructs. The maximum Mahalanobis distance was 24.37, which remained below the conservative χ^2^ threshold of 27.88 for nine variables at *p* < 0.001. Therefore, no cases were removed because of outlier concerns.

Normality was assessed using skewness and kurtosis values for the composite variables. As reported in Table 4, the skewness and kurtosis values were within acceptable ranges for survey-based research. Since the study also used NCA, fsQCA, and machine-learning methods, strict normality was not required for every analytical stage. The distributional results nevertheless suggested that the dataset did not show serious non-normality.

Common method bias was addressed procedurally and statistically. Procedurally, the study used a two-wave design: the psychological and organizational conditions were measured in Wave 1, while AI disclosure silence was measured in Wave 2. This temporal separation reduced the risk of measuring all constructs at the same time. The survey was anonymous, participation was voluntary, respondents were informed that there were no right or wrong answers, and self-generated codes were used instead of personal identifiers. These procedures were intended to reduce evaluation concerns and same-time self-report bias.

Statistically, Harman’s single-factor test was conducted using the main survey items. The first unrotated factor explained 31.56% of the total variance, which was below the commonly used 50% threshold. This result suggested that common method bias was unlikely to dominate the dataset. 

Social desirability was considered because the study examined a potentially sensitive workplace behavior. Employees may hesitate to report that they conceal AI use if they believe this behavior could be viewed as unprofessional, unethical, or risky. A separate social desirability scale was not included in order to avoid survey fatigue. However, anonymity, neutral item wording, voluntary participation, and self-generated matching codes were used to reduce social desirability pressure. Response-pattern checks also showed no evidence of careless responding: no respondent gave the same answer across all main survey items, and no case showed extremely low response variation.

Table 6 summarizes the data-screening procedures and the suitability of the final dataset for the planned analyses.

The screening results supported the use of the final dataset in the planned analyses. The data contained complete matched two-wave responses, no invalid response values, no missing values in the main construct items, no serious outlier problems, acceptable distributional properties, and no clear indication of careless responding. However, because the study relies on self-reported survey data and does not include a separate social desirability scale, same-source bias and socially desirable responding cannot be fully ruled out. This issue is acknowledged in the limitations section.

### 4.5. Measurement Validation

Measurement validation was conducted before the main NCA, fsQCA, and explainable machine-learning analyses. Because AI disclosure silence is newly operationalized in this study, the validation process focused not only on reliability and model fit but also on whether AI disclosure silence could be empirically distinguished from conceptually related constructs such as fear of negative evaluation, perceived creativity threat, perceived job insecurity, psychological safety, and trust in management.

The validation process followed two stages. First, exploratory factor analysis was used to examine the initial dimensionality of the measurement items and to assess whether the AI disclosure silence items formed a distinct factor. This step was included to address the exploratory nature of the construct and to provide additional evidence regarding item structure. Second, confirmatory factor analysis was conducted to test the proposed nine-factor measurement model. Reliability was assessed using Cronbach’s alpha and composite reliability. Convergent validity was assessed through average variance extracted. Discriminant validity was examined using the Fornell–Larcker criterion and heterotrait–monotrait (HTMT) ratios. Model fit was evaluated using the comparative fit index (CFI), Tucker–Lewis index (TLI), root mean square error of approximation (RMSEA), and standardized root mean square residual (SRMR), following commonly used fit-index recommendations (Hu & Bentler, 1999).

Table 7 presents the reliability and convergent validity results for the nine constructs.

The reliability results supported the internal consistency of the constructs. Cronbach’s alpha values ranged from 0.875 to 0.929, exceeding the commonly accepted threshold of 0.70. Composite reliability values ranged from 0.914 to 0.946, indicating satisfactory construct reliability. AVE values ranged from 0.697 to 0.778, exceeding the 0.50 threshold and supporting convergent validity.

Confirmatory factor analysis supported the proposed nine-factor measurement structure. The model included AI disclosure silence, AI anxiety, fear of negative evaluation, perceived creativity threat, perceived job insecurity, psychological safety, AI policy clarity, AI/digital self-efficacy, and trust in management as separate latent constructs. Table 8 reports the model-fit indices used to evaluate the adequacy of this measurement structure.

The CFA results indicated an acceptable measurement model. The CFI value was above the preferred 0.95 level, while the TLI value was very close to 0.95 and above the general acceptability threshold. RMSEA and SRMR were both below recommended cut-off values, suggesting that the model did not show problematic misfit.

Discriminant validity was examined with particular care because several constructs are conceptually close. AI anxiety, fear of negative evaluation, perceived creativity threat, and perceived job insecurity may all reflect different forms of perceived threat, while psychological safety and trust in management both concern the perceived safety of disclosure. The Fornell–Larcker results showed that the square root of AVE for each construct was higher than its correlations with other constructs. HTMT values also remained below the conservative threshold of 0.85. These findings support the empirical distinction among the constructs.

The evidence is especially important for AI disclosure silence. Although the construct is conceptually related to defensive silence and other self-protective workplace behaviors, the measurement results suggest that it captures a distinct form of silence focused on the concealment of AI’s role in work-related outputs. The EFA and CFA results, together with reliability, convergent validity, and discriminant validity evidence, provide initial support for using AI disclosure silence as a separate construct in the present study. Since the construct is still emerging, further validation through pilot testing, expert review, cognitive interviews, and cross-sample replication remains necessary.

Table 9 presents the inter-construct correlation matrix and provides the initial relational pattern among the study variables.

AI disclosure silence was positively correlated with threat-based conditions, including AI anxiety, fear of negative evaluation, perceived creativity threat, and perceived job insecurity. The strongest positive correlations were observed with perceived creativity threat (r = 0.58), fear of negative evaluation (r = 0.56), and perceived job insecurity (r = 0.51), suggesting that employees reporting stronger threat perceptions also tended to report higher AI disclosure silence. In contrast, AI disclosure silence was negatively correlated with psychological safety, AI policy clarity, AI/digital self-efficacy, and trust in management. These negative correlations indicate that employees who perceived stronger safety, clearer AI guidance, higher AI-related capability, and greater managerial trust tended to report lower AI disclosure silence. The correlation values were moderate rather than excessively high, providing preliminary support that the constructs are related but not redundant.

### 4.6. Analytical Strategy

The analytical strategy was designed to examine AI disclosure silence from complementary but distinct perspectives. The study does not treat the methods as separate or competing techniques. Instead, they are used in a sequential logic: measurement validation, necessity assessment, configurational explanation, predictive modeling, and integrated interpretation. This sequence is appropriate because AI disclosure silence may not be associated with one isolated condition. It may emerge through different combinations of perceived threat, weak safety or governance conditions, and limited AI-related capability.

The analysis was conducted in five stages. First, the measurement structure was evaluated through exploratory factor analysis, confirmatory factor analysis, reliability tests, convergent validity, and discriminant validity checks. This stage was especially important because AI disclosure silence is newly operationalized in this study and needed to be distinguished from conceptually related constructs. Second, Necessary Condition Analysis was used to examine whether any psychological or organizational condition functioned as a necessary bottleneck for high AI disclosure silence. Third, fsQCA was applied to identify sufficient configurations associated with high AI disclosure silence. Fourth, fsQCA was also used to examine configurations associated with low AI disclosure silence, because the absence of silence may not simply be the reverse of its presence. Fifth, Random Forest, XGBoost, and SHAP analysis were used to assess model-based predictive relevance and to interpret how each condition contributed to the machine-learning model’s predictions.

No chemicals, reagents, laboratory devices, instruments, commercial cell lines, biological samples, or physical experimental materials were used in this behavioral survey study. All statistical and computational analyses were conducted using the software versions used in the analyses. Descriptive statistics, reliability analysis, exploratory factor analysis, correlation analysis, and data-screening procedures were conducted using IBM SPSS Statistics, version 30.0 (IBM Corp., Armonk, NY, USA). Confirmatory factor analysis was conducted using IBM SPSS Amos, version 26.0 (IBM Corp., Armonk, NY, USA). Necessary Condition Analysis was conducted using the NCA package, version 5.0.1, in R (R Foundation for Statistical Computing, Vienna, Austria). Fuzzy-set Qualitative Comparative Analysis was conducted using the QCA package, version 3.25, in R (R Foundation for Statistical Computing, Vienna, Austria). Machine-learning analyses were conducted using Python, version 3.14.6 (Python Software Foundation, Wilmington, DE, USA), with scikit-learn, version 1.9.0, XGBoost, version 3.1.3, and SHAP, version 0.52.0.

Table 10 summarizes the connection between the research questions and the analytical methods.

Each method answers a different question. NCA examines whether a condition must be present for high AI disclosure silence to become possible. fsQCA examines how conditions combine into alternative sufficient pathways. Machine-learning models assess predictive patterns in the data, while SHAP explains the contribution of each condition to the model’s predictions. The machine-learning results are therefore interpreted as predictive and explanatory at the model level, not as causal evidence about employee behavior.

The interpretation of the findings follows the same multi-stage logic. A condition that appears as necessary in NCA, core in fsQCA, and highly relevant in SHAP is treated as a particularly robust condition. A condition that appears only in fsQCA may still be meaningful as part of a specific pathway, even if it is not dominant in the predictive model. Similarly, a condition with high SHAP importance may have predictive relevance without functioning as a necessary bottleneck. This integrated interpretation helps avoid treating the methods as redundant and allows the study to distinguish between necessity, sufficiency, and model-based predictive relevance.

### 4.7. Necessary Condition Analysis

Necessary Condition Analysis was used to address RQ1 by examining whether any psychological or organizational condition functioned as a necessary bottleneck for high AI disclosure silence. NCA is suitable for this purpose because it follows a necessity logic rather than an average-effect logic. A condition may be necessary when high levels of the outcome cannot occur unless the condition reaches a minimum level. In this sense, a necessary condition is not interpreted as sufficient by itself; rather, it indicates a constraint that must be present for the outcome to become possible (Dul, 2016).

In this study, the outcome variable was Wave 2 AI disclosure silence. The candidate necessary conditions were the Wave 1 variables: AI anxiety, fear of negative evaluation, perceived creativity threat, perceived job insecurity, psychological safety, AI policy clarity, AI/digital self-efficacy, and trust in management. For protective conditions, the interpretation also considered the relevance of their absence. For example, low psychological safety, low AI policy clarity, low trust in management, or low AI/digital self-efficacy may be more relevant for high AI disclosure silence than their high levels.

The basic necessity logic can be expressed as follows:

(1) Y≤f(X)

where (Y) represents AI disclosure silence and (X) represents a possible necessary condition. This expression means that the maximum achievable level of AI disclosure silence may be constrained by the level of a given condition. For example, if fear of negative evaluation is a necessary condition, very high AI disclosure silence should not appear when fear of negative evaluation is very low.

The analysis used the ceiling envelopment-free disposal hull (CE-FDH) and ceiling regression-free disposal hull (CR-FDH) techniques. CE-FDH identifies the empirical ceiling boundary based on observed cases, while CR-FDH provides a smoother ceiling line. Using both techniques allowed the necessity interpretation to be assessed with greater caution. Necessity effect sizes were interpreted using the commonly applied benchmarks in NCA, where values around 0.10, 0.30, and 0.50 indicate small, medium, and large necessity effects, respectively (Dul, 2016). Conditions with very small effect sizes were not treated as substantively meaningful, even when statistically significant.

For conditions showing meaningful necessity effects, bottleneck tables were produced. These tables indicate the minimum level of a condition required for different target levels of AI disclosure silence. The bottleneck logic is useful because it translates necessity findings into practical threshold values. For instance, if a high level of AI disclosure silence requires a minimum level of perceived creativity threat, this suggests that creativity-related concerns may function as a lower-bound condition for strong concealment of AI use.

The NCA procedure followed four steps. First, composite scores were calculated by averaging the items belonging to each construct. Second, each Wave 1 condition was tested separately as a possible necessary condition for Wave 2 AI disclosure silence. Third, the ceiling effect size and statistical significance were examined for each condition. Fourth, bottleneck tables were generated for conditions with meaningful necessity evidence.

The interpretation of NCA results was based on both statistical and theoretical relevance. A condition was treated as meaningful only when it showed a substantive necessity effect and aligned with the theoretical logic of the study. NCA therefore provided a first analytical layer by identifying whether any condition constrained the possibility of high AI disclosure silence. The following fsQCA stage then examined how different conditions combined into sufficient configurations for high and low AI disclosure silence.

### 4.8. Fuzzy-Set Qualitative Comparative Analysis

Fuzzy-set Qualitative Comparative Analysis was used to address RQ2 and RQ4. The method was selected because AI disclosure silence may not be associated with one isolated condition. Instead, different combinations of threat-based, safety/governance, and capability-related conditions may be linked to the same outcome. fsQCA is suitable for this purpose because it examines configurational relationships, equifinality, conjunctural causation, and asymmetry (Ragin, 2008; Fiss, 2011). In this study, this means that one employee may remain silent because of creativity threat and fear of negative evaluation, while another may do so because of job insecurity, low trust, unclear AI policy, or weak AI self-efficacy.

Unlike regression-based approaches, fsQCA does not estimate the average net effect of each variable. It examines whether specific combinations of conditions are sufficient for an outcome. This logic fits the theoretical argument that AI disclosure silence may emerge through different psychological profiles rather than through a single universal pathway. The same logic was applied to low AI disclosure silence because the absence of silence may not simply be the reverse of its presence. Separate analyses were therefore conducted for high AI disclosure silence and for the negated outcome, low AI disclosure silence.

The fsQCA procedure was conducted using fsQCA 4.1. Composite scores were first calculated by averaging the items belonging to each construct. Since all constructs were measured using a seven-point Likert scale, the composite scores were calibrated into fuzzy-set membership scores ranging from 0 to 1. Following the direct calibration logic proposed by Ragin (2008), three qualitative anchors were used: 6 for full membership, 4 for the crossover point, and 2 for full non-membership. A value of 6 indicated that a respondent was mostly in the set, a value of 4 represented maximum ambiguity, and a value of 2 indicated that a respondent was mostly out of the set.

The calibration logic can be expressed as:

(2) $$X_{f}=calibrate(X;f,c,n)$$

where $X_{f}$ represents the calibrated fuzzy-set score, X represents the original composite score, f represents the full membership threshold, c represents the crossover point, and n represents the full non-membership threshold. In this study, the calibration anchors were f=6, c=4, and n=2.

After calibration, truth tables were constructed for high AI disclosure silence and low AI disclosure silence. The negated outcome was calculated as:

(3) ∼X=1−X

where ∼X represents membership in low AI disclosure silence and X represents membership in high AI disclosure silence. This step was necessary because fsQCA assumes causal asymmetry. Conditions associated with openness about AI use may differ from the conditions associated with concealment.

The truth tables were evaluated using frequency and consistency thresholds. Given the sample size of 635, a minimum frequency threshold of three cases per configuration was used to avoid interpreting rare combinations. A consistency threshold of 0.80 was applied to identify configurations sufficiently associated with the outcome. Consistency indicates the extent to which cases sharing a configuration also display the outcome, while coverage indicates the empirical relevance of the configuration.

The consistency and coverage formulas are:

(4) $$Consistency(X\leq Y)=\frac{\sum min(X_{i},Y_{i})}{\sum X_{i}}$$

(5) $$Coverage(X\leq Y)=\frac{\sum min(X_{i},Y_{i})}{\sum Y_{i}}$$

where $X_{i}$ represents the membership score of case i in a given configuration and $Y_{i}$ represents the membership score of case i in the outcome.

Three solution types were generated: complex, parsimonious, and intermediate solutions. The intermediate solution was used for the main interpretation because it balances empirical evidence and theoretical expectations. Core and peripheral conditions were identified by comparing the parsimonious and intermediate solutions. A condition was treated as core when it appeared in both solutions and as peripheral when it appeared only in the intermediate solution (Fiss, 2011).

The configurations were interpreted as theoretically meaningful pathways rather than as isolated technical results. For high AI disclosure silence, the expected pathways may reflect identity protection, insecurity-driven concealment, low-trust disclosure avoidance, or policy-ambiguity silence. For low AI disclosure silence, the configurations may reflect openness supported by psychological safety, clear AI policy, trust in management, and stronger AI self-efficacy. These labels were used to connect the fsQCA results to the theoretical argument instead of presenting the findings only as combinations of present and absent conditions.

The fsQCA stage contributed to the study by identifying alternative sufficient pathways associated with high and low AI disclosure silence. It complemented NCA by moving from bottleneck conditions to configurational sufficiency. While NCA shows whether a condition must be present for high silence to become possible, fsQCA shows how several conditions combine into distinct disclosure-silence profiles.

### 4.9. Explainable Machine Learning

Explainable machine learning was used to address RQ3 by assessing the model-based predictive relevance of the psychological and organizational conditions associated with AI disclosure silence. This stage complemented the NCA and fsQCA analyses. NCA examined whether any condition functioned as a necessary bottleneck, and fsQCA examined how conditions combined into sufficient configurations. The machine-learning stage focused on predictive patterns and on the relative contribution of each condition to the model’s predictions.

The outcome variable was the Wave 2 composite score of AI disclosure silence. The predictors were the Wave 1 composite scores of AI anxiety, fear of negative evaluation, perceived creativity threat, perceived job insecurity, psychological safety, AI policy clarity, AI/digital self-efficacy, and trust in management. The analysis was conducted in Python, version 3.14.6 (Python Software Foundation, Wilmington, DE, USA), using scikit-learn, version 1.9.0, XGBoost, version 3.1.3, and SHAP, version 0.52.0. The dataset was divided into training and testing subsets so that model performance could be evaluated on unseen data rather than only on the data used for model training.Two tree-based models were considered: Random Forest and extreme gradient boosting (XGBoost). Random Forest was selected because it can capture non-linear relationships and interaction patterns by combining multiple decision trees (Breiman, 2001). XGBoost was included because it uses gradient boosting to improve predictive accuracy and is widely used for structured tabular data (T. Chen & Guestrin, 2016). These models were appropriate because AI disclosure silence may be associated with complex and non-linear relationships among threat-based, safety/governance, and capability-related conditions.

Model performance was evaluated using standard regression indicators, including root mean squared error (RMSE), mean absolute error (MAE), and coefficient of determination (R^2^). These indicators were calculated on the test dataset to assess how well the trained models predicted AI disclosure silence for cases not used during model training. The better-performing and more stable model was then selected for SHAP-based interpretation.

SHapley Additive exPlanations (SHAP) analysis was used to interpret the model’s predictions. SHAP values, based on Shapley values from cooperative game theory, estimate how much each predictor contributes to a specific model prediction (Lundberg & Lee, 2017). In this study, SHAP was used to show how conditions such as fear of negative evaluation, perceived creativity threat, perceived job insecurity, psychological safety, AI policy clarity, AI/digital self-efficacy, and trust in management contributed to the predicted level of AI disclosure silence.

The SHAP interpretation was kept at the model level. A positive SHAP value indicates that a condition increases the predicted level of AI disclosure silence in the model, whereas a negative SHAP value indicates that a condition decreases the predicted level. This should not be interpreted as causal evidence that the condition directly produces employee silence. Instead, SHAP explains how the machine-learning model uses each condition when making predictions.

The machine-learning stage contributed a predictive and interpretable layer to the study. Conditions with high SHAP importance were interpreted as having stronger model-based predictive relevance. These findings were then compared with the NCA and fsQCA results. A condition that appeared as necessary in NCA, core in fsQCA, and highly important in SHAP was treated as a robust condition. A condition that appeared only in fsQCA was still considered meaningful if it formed part of a theoretically coherent pathway. In this way, the machine-learning results were used to complement, rather than replace, the necessity and configurational analyses.

### 4.10. Integration of Analytical Outputs

The final step was to read the results from NCA, fsQCA, and explainable machine learning together. This was necessary because each method provides a different type of evidence. NCA shows whether a condition works as a bottleneck for high AI disclosure silence. fsQCA shows how different conditions combine into meaningful pathways. SHAP-based interpretation shows which conditions contribute most strongly to the machine-learning model’s predictions. Taken together, these methods help provide a more complete understanding of why marketing employees may hide or disclose AI use.

The interpretation followed the logic of each method. A condition identified through NCA was treated as a possible minimum requirement. This means that high AI disclosure silence may be unlikely unless that condition reaches a certain level. A condition appearing as core in fsQCA was interpreted as part of a sufficient pathway. In other words, the condition may matter because of how it works together with other psychological or organizational factors. A condition with high SHAP importance was interpreted as having strong predictive relevance in the machine-learning model.

The findings were interpreted cautiously. When a condition appeared as important across NCA, fsQCA, and SHAP, it was treated as a robust condition associated with AI disclosure silence. When a condition appeared only in fsQCA, it was not dismissed. Such a condition may still be important within a specific pathway, even if it is not necessary on its own or dominant in the predictive model. Similarly, when a condition showed high SHAP importance but did not appear as necessary in NCA, it was interpreted as predictively relevant rather than as a bottleneck.

This integrated reading helped separate three forms of evidence. Necessity evidence indicates whether a condition limits the possibility of high AI disclosure silence. Configurational evidence shows how conditions come together to form disclosure-silence profiles. Predictive evidence shows which conditions the machine-learning model relies on most strongly when estimating AI disclosure silence. Keeping these forms of evidence separate helped avoid overstating the meaning of any single method.

The results were not interpreted as causal effects. The study uses a two-wave survey design, not an experimental design. For this reason, the combined findings are presented as evidence of associations, configurations, and model-based predictive relevance. This approach fits the purpose of the study: to understand AI disclosure silence as a complex post-adoption disclosure behavior shaped by perceived threat, workplace safety and governance, and AI-related capability.

## 5. Results

### 5.1. Overview of Analytical Results

This section presents the findings in the same order as the analytical strategy. The analysis first examines whether any condition works as a necessary bottleneck for high AI disclosure silence. It then moves to the configurational results, which show how different psychological and organizational conditions combine in pathways associated with high and low AI disclosure silence. Finally, the machine-learning and SHAP results show which conditions contributed most strongly to the model’s predictions.

The findings are interpreted cautiously. The study uses a two-wave survey design, which provides temporal separation between the explanatory conditions and AI disclosure silence, but it does not establish causality. For this reason, the results are discussed as necessity evidence, configurational evidence, and model-based predictive evidence rather than as causal effects.

The order of the results follows the logic of the research questions. NCA is used first because it shows whether high AI disclosure silence depends on any minimum condition. fsQCA then shows whether different combinations of threat-based, safety/governance, and capability-related conditions are linked to high or low AI disclosure silence. The machine-learning stage adds a predictive perspective by identifying which conditions the model relies on most strongly when estimating AI disclosure silence.

This sequence is important because AI disclosure silence is unlikely to have one single explanation. Some employees may hide AI use because they fear negative evaluation or feel that AI threatens their creativity. Others may remain silent because AI policies are unclear, trust in management is weak, or the workplace does not feel safe for discussing AI-supported work. The results therefore focus not only on which conditions matter but also on how they appear together in different disclosure-silence pathways.

### 5.2. Necessary Condition Analysis Results

Necessary Condition Analysis was conducted to answer RQ1 by examining whether any Wave 1 psychological or organizational condition functioned as a necessary bottleneck for high Wave 2 AI disclosure silence. The analysis included AI anxiety, fear of negative evaluation, perceived creativity threat, perceived job insecurity, low psychological safety, low AI policy clarity, low AI/digital self-efficacy, and low trust in management. For the protective variables, the low-condition forms were used because silence was expected to become more likely when psychological safety, policy clarity, self-efficacy, or trust were weak.

Table 11 presents the NCA results. The CE-FDH effect sizes ranged from 0.030 to 0.090, while the CR-FDH effect sizes ranged from 0.006 to 0.036. None of the conditions reached the practical necessity threshold of 0.10. Although several conditions were statistically significant, their effect sizes were too small to be interpreted as meaningful necessary conditions. Therefore, no single condition can be considered an indispensable prerequisite for high AI disclosure silence.

The strongest CE-FDH effect was observed for AI anxiety, followed by low trust in management, perceived creativity threat, perceived job insecurity, and fear of negative evaluation. However, even these values remained below 0.10. This means that high AI disclosure silence was not strongly constrained by any single psychological or organizational condition. In practical terms, employees could report high AI disclosure silence even when one particular condition, such as AI anxiety or fear of negative evaluation, was not extremely high.

Because no condition reached the practical threshold, the bottleneck table should be interpreted as exploratory rather than as evidence of strong necessity. Table 12 reports the minimum levels of the selected conditions required for different target levels of AI disclosure silence. The values are shown on the original seven-point scale. For low trust in management, higher values indicate weaker trust.

The bottleneck values further support the conclusion that no strong necessary condition was present. For example, reaching a 90% target level of AI disclosure silence required only a minimum AI anxiety score of 3.20, perceived creativity threat of 1.80, perceived job insecurity of 2.20, and low trust in management of 2.75. These values are relatively low on a seven-point scale. Fear of negative evaluation also remained low across the bottleneck table. This pattern suggests that high AI disclosure silence does not require any single condition to reach a very high level.

The NCA findings are theoretically useful despite the absence of a strong necessary condition. They show that AI disclosure silence is unlikely to depend on one indispensable psychological trigger. Employees may hide AI use for different reasons: some may feel anxious about AI, some may worry about creativity-related judgment, some may feel insecure about their jobs, and others may not trust how managers will respond. The absence of a strong bottleneck therefore supports the next stage of the analysis. If no single condition is necessary on its own, the more relevant question becomes how different conditions combine into pathways associated with high or low AI disclosure silence.

### 5.3. fsQCA Results for High AI Disclosure Silence

The fsQCA analysis identified three configurations associated with high AI disclosure silence. These configurations show that employees may hide AI use through different psychological and organizational pathways. In all three configurations, AI anxiety, fear of negative evaluation, and low trust in management appeared as core conditions. Table 13 presents these high-silence configurations and reports their consistency and coverage values. The solution consistency was 0.934, and the solution coverage was 0.403.

The first configuration, labelled the generalized threat and trust-loss pathway, combines high AI anxiety, high fear of negative evaluation, high perceived creativity threat, high perceived job insecurity, and low trust in management. This pathway suggests that employees may hide AI use when AI is experienced as a broad professional threat. In this profile, disclosure may feel risky because it can raise concerns about competence, creativity, job security, and managerial judgment at the same time. Low psychological safety and low policy clarity were not part of this configuration, which means that the combination of serious psychological threat and low managerial trust was sufficient without these additional conditions.

The second configuration, labelled the evaluation-insecurity and weak-guidance pathway, combines high AI anxiety, high fear of negative evaluation, high perceived job insecurity, low psychological safety, low AI policy clarity, and low trust in management. This pathway points to a more organizationally fragile disclosure context. Employees in this profile may hide AI use not only because they feel anxious and fear negative judgment, but also because they do not experience the workplace as safe or clearly guided. Perceived creativity threat was not required in this configuration, indicating that AI disclosure silence may occur even without strong concerns about creative identity when job insecurity, weak guidance, and low trust are present together.

The third configuration, labelled the creativity-threat and weak-safety pathway, combines high AI anxiety, high fear of negative evaluation, high perceived creativity threat, low psychological safety, low AI policy clarity, and low trust in management. This pathway is especially relevant for marketing work because it links AI disclosure silence to concerns about creativity, originality, and professional image. Employees may remain silent when they believe that disclosing AI use could make their work appear less creative or less personally valuable. In this configuration, job insecurity was not required, suggesting that employees may hide AI use even when job loss is not the central concern. The stronger issue is reputational and creative devaluation.

These results support the configurational logic of the study. High AI disclosure silence was not associated with one single condition alone. Instead, it appeared through different combinations of threat, weak safety, weak guidance, and low managerial trust. AI anxiety, fear of negative evaluation, and low trust in management were central across all three pathways, while perceived creativity threat and perceived job insecurity appeared in different profiles. This pattern suggests that some employees may hide AI use to protect their creative identity, while others may do so because they feel insecure about their job or uncertain about how managers will interpret AI-supported work.

### 5.4. fsQCA Results for Low AI Disclosure Silence

The fsQCA analysis also identified three configurations associated with low AI disclosure silence. These configurations answer RQ4 by showing that openness about AI use is not simply the opposite of concealment. Table 14 presents the low-silence configurations and reports their consistency and coverage values. Low AI disclosure silence was linked not only to lower threat perceptions but also to supportive workplace conditions such as clear AI policy, trust in management, psychological safety, and AI/digital self-efficacy. The solution consistency was 0.940, and the solution coverage was 0.388.

The first configuration, labelled the low-threat and trust pathway, combines low AI anxiety, low fear of negative evaluation, low perceived creativity threat, low perceived job insecurity, high AI policy clarity, and high trust in management. This pathway suggests that employees are less likely to hide AI use when they do not view AI as a threat to competence, creativity, or job security. At the same time, clear organizational guidance and managerial trust appear to make disclosure feel safer and more legitimate.

The second configuration, labelled the safe and capable disclosure pathway, combines low AI anxiety, low fear of negative evaluation, low perceived creativity threat, high psychological safety, high AI policy clarity, high AI/digital self-efficacy, and high trust in management. This pathway shows that openness about AI use is not based only on the absence of fear. Employees also need to feel safe, capable, and supported. In this profile, employees may be more comfortable explaining how they used AI because they trust their managers, understand the rules, and feel confident in their own AI-related competence.

The third configuration, labelled the security and support pathway, combines low AI anxiety, low fear of negative evaluation, low perceived job insecurity, high psychological safety, high AI policy clarity, high AI/digital self-efficacy, and high trust in management. This pathway indicates that employees are less likely to conceal AI use when AI does not feel like a job-security threat and when the workplace provides supportive disclosure conditions. In this configuration, perceived creativity threat was not required, suggesting that low AI disclosure silence can still occur even when creativity concerns are not central, provided that job-security concerns are low and organizational support is strong.

These findings support the asymmetric logic of fsQCA. The configurations associated with low AI disclosure silence were not simple mirror images of the high-silence pathways. High AI disclosure silence was mainly shaped by AI anxiety, fear of negative evaluation, low trust, and either creativity threat or job insecurity. By contrast, low AI disclosure silence required more than reduced threat. It was also associated with positive disclosure conditions: clear AI policy, managerial trust, psychological safety, and AI/digital self-efficacy. This suggests that reducing silence in marketing work is not only about lowering anxiety or fear. Organizations also need to create a setting where employees feel safe, guided, trusted, and capable enough to discuss AI-supported work openly.

### 5.5. Machine-Learning Model Performance

Machine-learning analysis was conducted to assess how well the Wave 1 psychological and organizational conditions predicted Wave 2 AI disclosure silence. The predictors included AI anxiety, fear of negative evaluation, perceived creativity threat, perceived job insecurity, psychological safety, AI policy clarity, AI/digital self-efficacy, and trust in management. The dataset was divided into an 80/20 training–test split. Accordingly, 508 cases were used for model training, and 127 cases were reserved for test-set evaluation. 

Two tree-based models were compared: Random Forest and XGBoost. The purpose of this stage was not to replace the NCA and fsQCA findings, but to add a predictive layer to the analysis. While NCA examined whether any condition functioned as a bottleneck and fsQCA identified sufficient pathways, the machine-learning stage examined which model produced stronger predictive performance on unseen data. Table 15 reports the training and test performance of the two models.

The results show that both models predicted AI disclosure silence with moderate accuracy. Random Forest performed better on the training set, with an R^2^ value of 0.847, but its test-set performance was slightly weaker. XGBoost showed more balanced generalization, with a test-set root mean squared error (RMSE) of 1.125, mean absolute error (MAE) of 0.854, and coefficient of determination (R^2^) of 0.427. This means that XGBoost explained approximately 42.7% of the variance in AI disclosure silence in the unseen test data. For this reason, XGBoost was selected for the SHAP-based interpretation. 

A five-fold cross-validation check was also conducted to examine whether the model performance was stable. The cross-validation results showed that Random Forest and XGBoost performed similarly. Random Forest produced a mean cross-validated RMSE of 1.102 and mean R^2^ of 0.472, while XGBoost produced a mean cross-validated RMSE of 1.114 and mean R^2^ of 0.461. These results suggest that the predictive findings were not dependent on a single train–test split or model specification.

The model-performance results should be interpreted as predictive evidence, not as causal evidence. The findings indicate that the Wave 1 conditions contained meaningful information for estimating later AI disclosure silence, but they do not show that any single condition directly caused employees to hide AI use. The next section uses SHAP analysis to interpret how the selected model used each condition when making predictions.

### 5.6. SHAP-Based Predictor Interpretation

SHAP analysis was used to interpret how the selected XGBoost model used each condition when predicting AI disclosure silence. The purpose of this stage was not to identify causal effects, but to make the machine-learning results more transparent. Higher mean absolute SHAP values indicate that a condition contributed more strongly to the model’s predictions. Table 16 ranks the predictors according to their SHAP-based contribution to the model.

**Figure 2:**
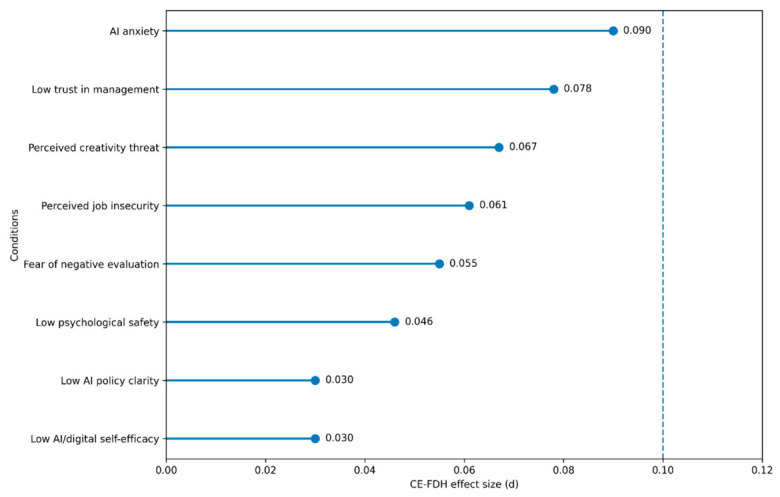
Necessary Condition Effect Sizes for AI Disclosure Silence.

Figure 2 presents the SHAP summary plot for the selected XGBoost model. The figure shows how each condition contributed to the model’s predictions of AI disclosure silence. Higher SHAP values indicate a stronger contribution to higher predicted AI disclosure silence, while lower SHAP values indicate a contribution to lower predicted silence.

The SHAP pattern confirms that fear of negative evaluation, AI anxiety, perceived creativity threat, and trust in management were the most influential predictors in the model. These results are consistent with the fsQCA findings, where these conditions appeared as central elements in the high-silence configurations.

Fear of negative evaluation had the strongest model-based predictive relevance. This suggests that the XGBoost model relied most heavily on whether employees expected negative judgment from managers, colleagues, or clients when estimating AI disclosure silence. This finding fits the marketing-work context because creative outputs are often evaluated subjectively. If employees believe that AI-supported work may be judged as less original, less effortful, or less professionally valuable, they may become less willing to disclose AI use.

AI anxiety was the second strongest predictor in the SHAP analysis. Higher AI anxiety increased the predicted level of AI disclosure silence in the model. This indicates that employees who feel uneasy about AI’s implications for marketing work may be more likely to conceal AI use. The result does not mean that anxiety causes silence, but it shows that the model treated AI anxiety as an important condition when estimating later disclosure silence.

Perceived creativity threat ranked third. This result is central to the theoretical argument of the study. In marketing work, employees often build their professional image around creativity, originality, and strategic contribution. When AI use is perceived as weakening the human value of creative work, employees may prefer to keep AI’s role invisible. This supports the idea that AI disclosure silence is not only about technology anxiety; it is also about protecting creative identity and professional legitimacy.

Trust in management ranked fourth and showed a protective pattern. Higher trust in management decreased the predicted AI disclosure silence in the model. This means that when employees believed managers would respond fairly and constructively to AI disclosure, the model estimated lower levels of silence. This finding strengthens the governance side of the framework. Disclosure appears less risky when employees trust that managers will not interpret AI use as laziness, incompetence, or lack of originality.

AI policy clarity also decreased predicted AI disclosure silence. Although its SHAP value was lower than fear of negative evaluation, AI anxiety, creativity threat, and trust, it still contributed meaningfully to the model. Clear rules about when AI can be used, when disclosure is expected, and how AI-supported outputs should be checked may reduce uncertainty around AI use. In this sense, policy clarity not only regulates AI behavior; it also helps employees understand whether disclosure is safe and appropriate.

Perceived job insecurity had moderate predictive relevance. Higher job insecurity increased predicted AI disclosure silence, suggesting that employees may be more likely to hide AI use when they believe AI could reduce their job stability or professional value. Psychological safety and AI/digital self-efficacy had lower SHAP values, but their directions remained theoretically meaningful. Higher psychological safety and stronger AI/digital self-efficacy were associated with lower predicted silence, indicating that employees may be more willing to disclose AI use when they feel safe discussing it and confident in their ability to use AI responsibly.

The SHAP results complement the fsQCA findings. Fear of negative evaluation, AI anxiety, perceived creativity threat, and trust in management appeared as important predictive conditions and were also central to the high-silence configurations. This convergence suggests that AI disclosure silence is shaped by a combination of evaluative concern, AI-related uncertainty, creative identity threat, and managerial trust. At the same time, differences between SHAP and fsQCA should not be treated as contradictions. SHAP reflects model-based predictive relevance across the dataset, whereas fsQCA identifies specific condition combinations. A condition may therefore have modest SHAP importance but still matter within a particular disclosure-silence pathway.

### 5.7. Integrated Interpretation of NCA, fsQCA, and SHAP Findings

The three analytical stages provide complementary evidence about AI disclosure silence. The NCA results showed that no single condition reached the practical threshold for being a necessary condition. This means that high AI disclosure silence does not depend on one indispensable factor. Employees may hide AI use for different reasons, and no single psychological or organizational condition appears to be required in every case.

The fsQCA results clarified this point by showing that high AI disclosure silence emerged through different configurations. AI anxiety, fear of negative evaluation, and low trust in management appeared as core conditions across the high-silence pathways. However, perceived creativity threat, perceived job insecurity, low psychological safety, and low AI policy clarity played different roles across the configurations. This suggests that AI disclosure silence is not a uniform behavior. For some employees, it may reflect concern over creativity and originality. For others, it may reflect job insecurity, weak guidance, or low managerial trust.

The SHAP results added a predictive layer to this interpretation. Fear of negative evaluation, AI anxiety, perceived creativity threat, and trust in management had the strongest model-based predictive relevance. This pattern is consistent with the fsQCA findings because the same conditions appeared as central parts of the high-silence configurations. In particular, fear of negative evaluation and perceived creativity threat support the identity-protection logic of the study, while trust in management supports the safety and governance side of the framework.

The absence of strong NCA effects is not inconsistent with the fsQCA and SHAP results. NCA asks whether a condition is indispensable. fsQCA asks how conditions combine into sufficient pathways. SHAP shows which conditions the machine-learning model relies on most strongly when estimating AI disclosure silence. A condition may therefore be highly relevant in SHAP or central in fsQCA without being necessary in the NCA sense. This distinction is important because it shows why the methods should be read together rather than compared as if they answered the same question.

The combined findings suggest that AI disclosure silence is best understood as a multi-pathway post-adoption behavior. It is not simply the result of AI anxiety, nor is it only the result of unclear AI policy. Instead, it appears to form when employees experience AI-supported work as evaluatively risky, professionally sensitive, or insufficiently protected by managerial trust and organizational guidance. The findings also show that reducing AI disclosure silence may require more than lowering threat perceptions. Table 17 summarizes the main evidence from NCA, fsQCA, machine learning, and SHAP to show how the analytical results converge and differ.

### 5.8. Robustness and Sensitivity Checks

Several robustness and sensitivity checks were conducted to examine whether the main findings were stable across analytical decisions. These checks focused on predictive model comparison, cross-validation performance, configurational asymmetry, integration across analytical methods, and measurement validation. Table 18 summarizes the checks and their interpretation.

The predictive checks showed that the results were not dependent on a single model. Random Forest showed stronger training-set performance, but XGBoost provided slightly better test-set performance, with a test-set R^2^ of 0.427, RMSE of 1.125, and MAE of 0.854. For this reason, XGBoost was selected for SHAP interpretation. The five-fold cross-validation results also showed comparable model stability. Random Forest produced a mean cross-validated RMSE of 1.102 and a mean R^2^ of 0.472, while XGBoost produced a mean cross-validated RMSE of 1.114 and a mean R^2^ of 0.461.

The configurational checks supported the asymmetry assumption. The low-silence configurations were not simple reversals of the high-silence configurations. High AI disclosure silence was mainly associated with threat, fear of negative evaluation, creativity-related risk, job insecurity, and low trust. Low AI disclosure silence, by contrast, was associated with supportive conditions such as trust in management, clear AI policy, psychological safety, and AI/digital self-efficacy. This suggests that reducing silence requires more than lowering perceived threat; it also requires a disclosure environment that feels safe, clear, and fair.

The comparison across NCA, fsQCA, and SHAP further supported the main interpretation. NCA showed that no condition reached the practical necessity threshold, whereas fsQCA and SHAP showed that several conditions were still important in configurations or model-based prediction. This pattern is not contradictory because the methods answer different questions. NCA examines whether a condition is indispensable, fsQCA examines how conditions combine into pathways, and SHAP shows which variables contribute most strongly to model predictions.

The measurement validation checks also strengthened confidence in the findings. EFA, CFA, reliability, convergent validity, and discriminant validity results provided initial support for treating AI disclosure silence as a distinct construct. This is especially important because the construct is newly operationalized and conceptually close to defensive silence and other self-protective workplace behaviors. At the same time, these checks do not remove the need for further validation.

These robustness and sensitivity checks support the stability of the main interpretation within the present dataset. The findings were not dependent on a single predictive model, one train–test split, or one analytical logic. They suggest that AI disclosure silence is better understood as a multi-pathway behavior shaped by threat-based, safety/governance, and capability-related conditions. However, the checks do not establish causality. Future studies should test the model across other samples, sectors, cultures, and AI-governance contexts.

## 6. Discussion

The findings suggest that AI disclosure silence is best understood as a post-adoption disclosure behavior. The employees examined in this study were not avoiding AI or resisting its use. They were already using AI tools in their marketing work, but some were reluctant to reveal AI’s role in their outputs. This distinction is central to the contribution of the study. AI disclosure silence is not simply about whether employees accept AI. It concerns whether they feel safe, legitimate, and professionally protected enough to acknowledge AI-supported work.

The results show that AI disclosure silence does not depend on one single condition. The NCA findings indicated that no psychological or organizational condition reached the practical threshold for necessity. The fsQCA results then showed that high AI disclosure silence appeared through different pathways involving AI anxiety, fear of negative evaluation, creativity threat, job insecurity, low trust in management, weak psychological safety, and unclear AI policy. The SHAP results added a predictive layer by showing that fear of negative evaluation, AI anxiety, perceived creativity threat, and trust in management contributed most strongly to the machine-learning model’s predictions. Taken together, these findings suggest that employees may hide AI use for different reasons, but these reasons often involve perceived professional risk, social judgment, and uncertainty about managerial response.

### 6.1. Main Interpretation of the Findings

The study shows that hidden AI use is not necessarily a sign of unethical intent or resistance to organizational rules. In many cases, AI disclosure silence may reflect uncertainty about how AI-supported work will be evaluated. Employees may worry that disclosing AI use will make their work appear less original, less effortful, or less valuable. This concern is particularly relevant in marketing, where employees are often judged according to creativity, originality, persuasion, and strategic judgment. When AI contributes to slogans, campaign ideas, social media content, customer insights, or visual concepts, employees may become uncertain about how much credit will remain attached to their own professional contribution.

The absence of a strong necessary condition is theoretically meaningful. It indicates that AI disclosure silence is not built around one indispensable trigger. Employees do not need to experience only AI anxiety, only job insecurity, or only low trust in management to remain silent. Instead, AI disclosure silence appears to be shaped by different combinations of perceived threat and weak disclosure support. This explains why the configurational findings are important. The fsQCA results showed several pathways to high silence, including pathways based on generalized threat, evaluation insecurity, creativity threat, weak guidance, and low trust.

The SHAP results supported this interpretation by showing that fear of negative evaluation had the strongest model-based predictive relevance. This finding suggests that employees’ concern about how others will judge AI-supported work is central to understanding AI disclosure silence. AI anxiety and perceived creativity threat were also highly relevant, indicating that the issue is not only about technical uncertainty. It is also about how AI affects professional identity, creative ownership, and the perceived value of human contribution. Trust in management also played an important role. When employees believe managers will respond fairly to AI disclosure, silence becomes less likely in the predictive model.

The findings also show that reducing AI disclosure silence is not simply the reverse of increasing it. The low-silence configurations were not mirror images of the high-silence configurations. Lower anxiety and lower fear matter, but openness about AI use also appears to require positive workplace conditions. Clear AI policy, trust in management, psychological safety, and AI/digital self-efficacy were associated with pathways to lower silence. This suggests that organizations cannot reduce hidden AI use only by telling employees not to be afraid. They need to create a workplace environment where AI use can be discussed without punishment, embarrassment, or unfair judgment.

### 6.2. Theoretical Implications

This study contributes first to the employee silence literature by identifying a more specific form of silence in AI-supported work. Traditional employee silence usually refers to withholding opinions, concerns, suggestions, or information that could be useful for organizational learning and change (Morrison & Milliken, 2000; Van Dyne et al., 2003). AI disclosure silence is related to this literature, especially to defensive silence, but its object of concealment is different. Employees are not only withholding an opinion or concern. They are withholding information about the technological process behind a visible work output. The silence concerns AI-supported authorship, contribution, and production.

This distinction helps clarify why AI disclosure silence should be treated as a distinct but still emerging construct. It occurs after AI use has already taken place. It does not describe AI non-use, AI avoidance, or AI resistance. An employee may actively use AI, benefit from it, and still avoid disclosing its role. Although AI disclosure silence shares a self-protective logic with defensive silence, it differs because the concealed object is not an opinion, suggestion, or concern, but the role of AI in producing a visible work output. It also differs from knowledge hiding because the employee is not necessarily withholding requested expertise from others; rather, the employee conceals the technological contribution behind their own completed work. The construct therefore applies only when AI has actually been used and the employee intentionally withholds AI’s role in the work process or output. The findings should be read as initial support for the construct’s usefulness, not as evidence that its measurement is fully settled.

The study also contributes to workplace AI and human–AI collaboration research. Much of the discussion around workplace AI focuses on adoption, acceptance, trust in AI, resistance, or usage intention. The present findings shift attention to what happens after adoption. The question is no longer only whether employees use AI, but whether they can safely acknowledge AI’s role in their work. This point is consistent with recent studies showing that employee responses to intelligent technologies depend on intentions, identity, perceived insecurity, and organizational conditions (Wu et al., 2025a; Wu et al., 2025b; Xu et al., 2025; Nguyen et al., 2026). AI disclosure silence therefore adds a post-adoption layer to workplace AI research.

The findings also extend Conservation of Resources Theory. From this perspective, employees seek to protect valued resources such as professional reputation, competence image, job security, social acceptance, and creative identity (Hobfoll, 1989). AI disclosure may be interpreted as a possible resource-loss event. Employees may fear that revealing AI use will reduce the perceived originality of their work, weaken their professional legitimacy, or signal that parts of their role are replaceable. The high-silence configurations support this logic because creativity threat, job insecurity, AI anxiety, and fear of negative evaluation appeared in different pathways to silence.

The study further extends Psychological Safety Theory. Psychological safety is usually discussed in relation to speaking up, admitting mistakes, asking questions, or sharing concerns (Edmondson, 1999). The present findings suggest that AI disclosure can also be an interpersonal risk. Employees may not know whether managers or colleagues will interpret AI use as responsible innovation, efficient work practice, lack of effort, or reduced creativity. In this sense, psychological safety in AI-supported work is not only about expressing ideas or concerns. It also involves feeling safe enough to explain how AI contributed to one’s work.

The configurational contribution is also important. The findings show that AI disclosure silence is not a linear or single-factor behavior. The absence of a strong necessary condition, the presence of multiple fsQCA pathways, and the SHAP-based predictor rankings together suggest that AI disclosure silence is better understood as a multi-pathway phenomenon. Some pathways are more strongly identity-based, while others are more strongly insecurity-based or governance-based. This helps explain why general policies encouraging AI use may not be enough. Employees’ willingness to disclose AI use depends on how threat, safety, trust, policy clarity, and capability come together in their work environment.

### 6.3. Practical Implications

The findings have several implications for organizations that expect employees to use AI responsibly. First, organizations need to build an AI transparency culture. Employees should not feel that any disclosure of AI use will automatically be interpreted as laziness, cheating, or lack of originality. If AI use is informally encouraged but formally ambiguous, employees may choose silence as a safer option. Organizations should therefore communicate that responsible AI disclosure is part of accountability and quality control, not a confession of wrongdoing.

Second, organizations should create clear AI disclosure guidelines. Employees need to know which AI tools are acceptable, which tasks can be supported by AI, when disclosure is required, how AI-generated content should be checked, and who remains responsible for final outputs. This is especially important in marketing because AI-supported work may affect brand reputation, customer communication, copyright issues, data privacy, and factual accuracy. These guidelines should also be adapted to the organization’s culture, client expectations, regulatory setting, and level of AI-governance maturity.

Third, managers should be trained to respond to AI disclosure in a non-punitive and constructive way. The findings show that trust in management is closely connected to AI disclosure silence. If employees expect managers to interpret AI use as reduced effort or weak competence, they may hide it. Managers should therefore ask how AI was used, how outputs were checked, what human judgment was added, and whether any risks were considered. Such a response frames disclosure as part of professional responsibility rather than as a reason for blame.

Fourth, organizations should clarify authorship and originality norms for AI-supported work. Employees in creative and marketing roles may worry that AI use will weaken their professional identity. To reduce this concern, organizations can define what counts as acceptable AI assistance and what still requires human creativity, judgment, and accountability. AI-assisted originality standards can help employees understand how to combine AI-generated suggestions with their own expertise without feeling that their creative value is being erased.

Fifth, AI training should go beyond tool use. Many training programs focus on prompts, productivity, or technical features. The findings suggest that training should also include disclosure expectations, verification practices, bias checking, data confidentiality, copyright awareness, and responsible communication of AI-supported work. Employees with stronger AI/digital self-efficacy may feel more capable of explaining how they used AI and why the final output remains professionally valid. Training should therefore support both technical capability and disclosure confidence.

Finally, organizations should treat hidden AI use as a governance blind spot. When AI’s role remains invisible, organizations may not be able to evaluate the quality, accuracy, ethical risks, or accountability of AI-supported outputs. This does not mean that every minor AI interaction must be reported in detail. Rather, organizations should identify the situations in which disclosure matters most, especially when AI affects client-facing content, strategic decisions, customer data, legal exposure, or brand-sensitive communication.

### 6.4. Limitations and Future Research

Several limitations should be acknowledged. First, the study relied on self-reported survey data. The two-wave design reduced, but did not eliminate, same-source and self-report concerns. The topic is also sensitive. Some respondents may have underreported their tendency to conceal AI use because such behavior could be seen as unprofessional or ethically questionable. Although anonymity, neutral wording, and self-generated matching codes were used to reduce this risk, social desirability cannot be fully ruled out.

Second, the study does not support causal claims. The two-wave design is stronger than a one-shot cross-sectional survey, but it is not equivalent to an experimental or full longitudinal causal design. The findings should therefore be interpreted as associations, configurations, and model-based predictive patterns. Future research could use experiments, diary studies, longer panel designs, or field interventions to examine how changes in AI policy, managerial response, or psychological safety shape AI disclosure behavior over time.

Third, AI disclosure silence is newly operationalized in this study. The findings provide initial empirical support for the construct, but they should not be interpreted as final scale validation. Future research should strengthen the scale-development process through expert review, pilot testing, cognitive interviews, test–retest reliability, and cross-sample validation. Recent AI-context psychometric research also highlights the value of rigorous item development and validation when introducing new constructs in AI-enhanced settings (Ayasrah et al., 2026). Future studies should also compare AI disclosure silence directly with defensive silence, knowledge hiding, AI avoidance, and AI resistance to further test discriminant validity.

Fourth, the sample was recruited through professional networks using a non-probability sampling approach. Therefore, the sample should not be interpreted as statistically representative of the full marketing workforce. AI disclosure norms may differ depending on industry, country, organizational culture, managerial attitudes, and AI-governance maturity. Future studies should test the framework in larger and more diverse samples, including organizations with formal AI policies and organizations where AI use remains informal or ambiguous.

Fifth, the study focused on marketing employees. Marketing is a theoretically useful context because creativity, originality, authorship, and client-facing outputs are central to professional evaluation. However, AI disclosure silence may also appear in other forms of knowledge work, including design, consulting, education, software development, journalism, research, and professional services. Future research should examine whether the same pathways appear in these contexts or whether different professions create different disclosure risks.

Finally, the study examined individual employee perceptions. Organizational conditions such as psychological safety, trust in management, and AI policy clarity were measured through employee perceptions rather than objective organizational audits. Future research could combine employee surveys with policy document analysis, manager interviews, behavioral disclosure data, or organizational AI-governance assessments. Such designs would provide a richer understanding of how formal rules, informal norms, and managerial practices shape employees’ willingness to disclose AI-supported work.

## 7. Conclusions

This study examined AI disclosure silence as an emerging post-adoption disclosure behavior among marketing employees who already use AI tools in their work. The central issue was not whether employees accept or reject AI, but whether they feel able to disclose AI’s role in producing work-related outputs. AI disclosure silence should therefore be understood not as simple resistance to AI, but as a disclosure dilemma that emerges when employees use AI while remaining uncertain about how AI-supported work will be judged.

The findings show that AI disclosure silence is not explained by one single condition. No psychological or organizational condition functioned as a strong, necessary bottleneck. Instead, the results point to multiple pathways through which employees may conceal AI use. Fear of negative evaluation, AI anxiety, perceived creativity threat, perceived job insecurity, low trust in management, weak psychological safety, and unclear AI policy appeared in different configurations associated with high AI disclosure silence. The machine-learning results further indicated that fear of negative evaluation, AI anxiety, perceived creativity threat, and trust in management had strong model-based predictive relevance. These findings suggest that AI disclosure silence is shaped by both perceived threat and the quality of the organizational disclosure environment.

The study contributes to the employee silence and workplace AI literature by clarifying a distinct form of silence related to AI-supported work. Unlike AI avoidance or AI resistance, AI disclosure silence occurs after AI has already been used. Unlike general employee silence, it concerns the hidden role of AI in producing visible work outputs. This distinction is especially relevant in marketing, where creativity, originality, authorship, and professional judgment are central to how work is evaluated. The findings provide initial support for treating AI disclosure silence as a distinct but still developing construct.

The practical message is that organizations should not assume that AI use will automatically be disclosed simply because AI tools are available or encouraged. Employees may remain silent if disclosure feels professionally risky, unclear, or unsafe. Organizations therefore need to develop AI transparency cultures supported by clear disclosure guidelines, non-punitive managerial responses, responsible AI-use training, and explicit norms for AI-assisted authorship and originality. Such practices can help employees discuss AI-supported work openly while maintaining accountability, quality, and professional legitimacy.

The study also has important boundaries. The findings should be interpreted as associations, configurations, and model-based predictive patterns rather than causal effects. AI disclosure silence was newly operationalized, and further validation is needed through expert review, pilot testing, cognitive interviews, cross-sample replication, and comparison with related constructs such as defensive silence, knowledge hiding, AI avoidance, and AI resistance. Future research should also test the framework in other sectors, cultures, and organizational AI-governance contexts. As AI becomes increasingly embedded in knowledge work, understanding when employees disclose or conceal AI use will remain important for responsible, transparent, and psychologically safe AI integration.

## Figures and Tables

**Table 1:** Conceptual Distinction Between AI Disclosure Silence and Related Constructs.

Construct	Primary Object of Concealment or Response	Timing in Relation to AI Use	Core Behavioral Logic	Key Distinction from AI Disclosure Silence
AI disclosure silence	The use, role, or contribution of AI tools in a visible work output	After AI has already been used	Post-adoption concealment to protect legitimacy, authorship, creativity image, or evaluation security	Focal construct: employees use AI but withhold AI’s contribution to their own completed work
Defensive silence	Opinions, concerns, ideas, or information that may create personal risk	Not tied to AI use	Self-protective withholding to avoid negative consequences	Broader voice/silence behavior; not specifically about hidden AI-supported authorship or production
Knowledge hiding	Knowledge or expertise requested by another person	Not tied to AI use	Intentional withholding of requested knowledge	Concerns interpersonal knowledge withholding, not concealment of AI’s role in one’s own output
AI avoidance/resistance	Use or acceptance of AI itself	Before or during adoption	Non-use, opposition, reluctance, or rejection of AI	AI disclosure silence occurs after adoption; the employee may actively use AI while concealing it

Note. AI = artificial intelligence.

**Table 2:** Sample Characteristics and AI-Use Profile.

Category	Group	*n*	%
Gender	Female	316	49.8
	Male	297	46.8
	Prefer not to say	22	3.5
Age	18–24	81	12.8
	25–34	260	40.9
	35–44	187	29.4
	45–54	81	12.8
	55 or above	26	4.1
Education level	High school	28	4.4
	Associate degree	55	8.7
	Bachelor’s degree	343	54.0
	Master’s degree	184	29.0
	Doctorate	25	3.9
Position level	Intern/trainee	34	5.4
	Junior employee	110	17.3
	Specialist	169	26.6
	Senior specialist	156	24.6
	Manager	113	17.8
	Senior manager	46	7.2
	Director or above	7	1.1
Total work experience	Less than 1 year	39	6.1
	1–3 years	139	21.9
	4–6 years	180	28.3
	7–10 years	149	23.5
	More than 10 years	128	20.2
Marketing experience	Less than 1 year	50	7.9
	1–3 years	146	23.0
	4–6 years	203	32.0
	7–10 years	142	22.4
	More than 10 years	94	14.8
Organization type	Advertising agency	115	18.1
	Digital marketing agency	128	20.2
	Corporate marketing department	185	29.1
	E-commerce company	113	17.8
	Media/creative agency	67	10.6
	Consulting company	27	4.3
Organization size	1–10 employees	50	7.9
	11–50 employees	138	21.7
	51–250 employees	212	33.4
	251–500 employees	114	18.0
	More than 500 employees	121	19.1
AI-use frequency	A few times a month	108	17.0
	A few times a week	210	33.1
	Almost every day	205	32.3
	Several times a day	112	17.6

Note. AI = artificial intelligence; N = sample size; n = number of participants. Percentages may not total exactly 100 because of rounding.

**Table 3:** AI Tools and AI-Supported Marketing Tasks Reported by Participants.

Category	Item	*n*	%
AI tools used	Jasper	246	38.7
	Microsoft Copilot	243	38.3
	Canva AI	229	36.1
	Midjourney	224	35.3
	Gemini	216	34.0
	ChatGPT	215	33.9
AI-supported marketing tasks	Presentation preparation	242	38.1
	Campaign idea generation	230	36.2
	Visual design support	228	35.9
	Market research	223	35.1
	Advertising copy	220	34.6
	Social media posts	218	34.3
	Email/newsletter writing	215	33.9
	Customer analysis	209	32.9
	Content writing	207	32.6
	SEO content	201	31.7

Note. AI = artificial intelligence; n = number of responses; SEO = search engine optimization. Participants could select more than one AI tool and more than one AI-supported marketing task; therefore, percentages do not sum to 100.

**Table 4:** Descriptive Statistics of Main Constructs.

Construct	Wave	Mean	SD	Min	Max
AI anxiety	Wave 1	4.00	1.18	1.00	7.00
Fear of negative evaluation	Wave 1	3.96	1.20	1.00	7.00
Perceived creativity threat	Wave 1	3.88	1.15	1.00	7.00
Perceived job insecurity	Wave 1	4.02	1.22	1.00	7.00
Psychological safety	Wave 1	4.25	1.10	1.00	7.00
AI policy clarity	Wave 1	4.14	1.13	1.00	7.00
AI/digital self-efficacy	Wave 1	4.28	1.08	1.00	7.00
Trust in management	Wave 1	4.17	1.11	1.00	7.00
AI disclosure silence	Wave 2	3.92	1.19	1.00	7.00

Note. AI = artificial intelligence; N = sample size; SD = standard deviation. All variables were measured on a seven-point Likert scale.

**Table 5:** Measurement Constructs, Sources, and Sample Items.

Construct	Wave	Code	No. of Items	Source/Adaptation Basis	Sample Item
AI disclosure silence	Wave 2	W2_ADS	6	Adapted from organizational silence and employee silence literature (Morrison & Milliken, 2000; Van Dyne et al., 2003)	“I avoid telling my manager when I use AI tools for marketing-related tasks.”
AI anxiety	Wave 1	W1_AIA	5	Adapted from the Artificial Intelligence Anxiety Scale (Wang & Wang, 2022)	“I feel anxious when I think about how AI may change marketing jobs.”
Fear of negative evaluation	Wave 1	W1_FNE	5	Adapted from the Brief Fear of Negative Evaluation Scale (Leary, 1983)	“I worry that others will judge me negatively if they know I use AI at work.”
Perceived creativity threat	Wave 1	W1_PCT	5	Adapted from creative self-efficacy and creativity-related professional image logic (Tierney & Farmer, 2002)	“Using AI in marketing makes me feel that my creativity may be questioned.”
Perceived job insecurity	Wave 1	W1_PJI	5	Adapted from the Job Insecurity Scale (Vander Elst et al., 2014)	“I feel insecure about the future of my job because of AI developments.”
Psychological safety	Wave 1	W1_PS	6	Adapted from psychological safety research (Edmondson, 1999)	“In my team, it is safe to openly discuss the use of AI tools.”
AI policy clarity	Wave 1	W1_APC	6	Adapted from role clarity and role ambiguity logic (Rizzo et al., 1970)	“My organization clearly explains when AI tools may be used for marketing tasks.”
AI/digital self-efficacy	Wave 1	W1_AIDSE	5	Adapted from computer self-efficacy research (Compeau & Higgins, 1995)	“I am confident in my ability to use AI tools for marketing tasks.”
Trust in management	Wave 1	W1_TM	4	Adapted from organizational trust theory (Mayer et al., 1995)	“I trust my managers to respond fairly if I disclose AI use.”

Note. AI = artificial intelligence; W1 = Wave 1; W2 = Wave 2; ADS = AI disclosure silence; AIA = AI anxiety; FNE = fear of negative evaluation; PCT = perceived creativity threat; PJI = perceived job insecurity; PS = psychological safety; APC = AI policy clarity; AIDSE = AI/digital self-efficacy; TM = trust in management.

**Table 6:** Data Screening Results.

Screening Criterion	Procedure/Indicator	Result	Evaluation
Initial eligible Wave 1 responses	Respondents passing screening questions	691	Eligible starting pool
Final matched two-wave responses	Matched and complete Wave 1–Wave 2 records	635	Final dataset
Removed/unmatched responses	Incomplete, unmatched, or excluded cases	56	8.1% removal rate
Missing data	Missing values in main construct items	0	No missing-data problem
Valid response range	Likert-scale item range	1–7	All values within range
Duplicate respondent IDs	Duplicate self-generated IDs	0	No duplicate ID problem
Univariate outliers	Composite z-scores > ±3.29	0	No serious univariate outliers
Multivariate outliers	Mahalanobis distance, χ^2^ threshold = 27.88	Max = 24.37	No serious multivariate outliers
Normality: skewness	Composite-level skewness range	−0.174 to 0.090	Acceptable
Normality: kurtosis	Composite-level kurtosis range	−0.877 to −0.767	Acceptable
Common method bias	Harman’s single-factor test	First factor = 31.56%	Below 50%; acceptable
Straight-lining	Same response across all main items	0 cases	No straight-lining detected
Low response variation	Very low item-level variation pattern	0 cases	No serious response-pattern problem
Social desirability	Procedural controls and response-pattern review	No formal scale used	Controlled procedurally; noted as limitation

Note. AI = artificial intelligence; ID = identification; N = sample size; χ^2^ = chi-square.

**Table 7:** Reliability and Convergent Validity Results.

Construct	Code	Mean	Cronbach’s α	Composite Reliability	AVE	Evaluation
AI disclosure silence	W2_ADS	3.92	0.927	0.943	0.733	Acceptable
AI anxiety	W1_AIA	4.00	0.914	0.936	0.744	Acceptable
Fear of negative evaluation	W1_FNE	3.96	0.919	0.939	0.755	Acceptable
Perceived creativity threat	W1_PCT	3.88	0.914	0.936	0.745	Acceptable
Perceived job insecurity	W1_PJI	4.02	0.929	0.946	0.778	Acceptable
Psychological safety	W1_PS	4.25	0.913	0.933	0.697	Acceptable
AI policy clarity	W1_APC	4.14	0.920	0.938	0.715	Acceptable
AI/digital self-efficacy	W1_AIDSE	4.28	0.895	0.920	0.704	Acceptable
Trust in management	W1_TM	4.17	0.875	0.914	0.727	Acceptable

Note. AI = artificial intelligence; AVE = average variance extracted; N = sample size.

**Table 8:** Confirmatory Factor Analysis Model Fit Results.

Fit Index	Result	Recommended Threshold	Evaluation
CFI	0.956	≥0.90/preferably ≥ 0.95	Good fit
TLI	0.949	≥0.90/preferably ≥ 0.95	Acceptable to good fit
RMSEA	0.046	≤0.08/preferably ≤ 0.06	Good fit
SRMR	0.041	≤0.08	Good fit

Note. CFI = comparative fit index; TLI = Tucker–Lewis index; RMSEA = root mean square error of approximation; SRMR = standardized root mean square residual.

**Table 9:** Correlation Matrix of Main Constructs.

Construct	1	2	3	4	5	6	7	8	9
1. AI anxiety	1								
2. Fear of negative evaluation	0.44	1							
3. Perceived creativity threat	0.42	0.47	1						
4. Perceived job insecurity	0.46	0.43	0.45	1					
5. Psychological safety	−0.31	−0.36	−0.34	−0.33	1				
6. AI policy clarity	−0.28	−0.32	−0.31	−0.30	0.48	1			
7. AI/digital self-efficacy	−0.26	−0.29	−0.30	−0.27	0.42	0.44	1		
8. Trust in management	−0.30	−0.35	−0.33	−0.31	0.50	0.47	0.41	1	
9. AI disclosure silence	0.49	0.56	0.58	0.51	−0.43	−0.46	−0.39	−0.45	1

Note. AI = artificial intelligence; N = sample size. Correlations are based on composite scores.

**Table 10:** Analytical Strategy and Research Questions.

Research Question	Method	Analytical Purpose
RQ1: Which psychological and organizational conditions are necessary for high AI disclosure silence among marketing employees who use AI tools at work?	Necessary Condition Analysis	Identifies whether any condition functions as a necessary bottleneck for high AI disclosure silence
RQ2: Which combinations of threat-based, safety/governance, and capability-related conditions are sufficient for high AI disclosure silence in marketing work?	fsQCA	Identifies sufficient configurations associated with high AI disclosure silence
RQ3: Which conditions have the strongest model-based predictive relevance for AI disclosure silence among marketing employees?	Random Forest, XGBoost, and SHAP	Assesses predictive relevance and interprets how each condition contributes to model predictions
RQ4: Are the configurations associated with high AI disclosure silence different from those associated with low AI disclosure silence?	fsQCA for the negated outcome	Examines configurational asymmetry between high and low AI disclosure silence

Note. AI = artificial intelligence; RQ = research question; NCA = Necessary Condition Analysis; fsQCA = fuzzy-set Qualitative Comparative Analysis; SHAP = SHapley Additive exPlanations.

**Table 11:** NCA Results for AI Disclosure Silence.

Condition	Direction Used in NCA	CE-FDH Effect Size	CR-FDH Effect Size	*p*-Value	Interpretation
AI anxiety	High AI anxiety	0.090	0.035	0.002	Very small; below practical necessity threshold
Fear of negative evaluation	High fear of negative evaluation	0.055	0.011	0.002	Very small; below practical necessity threshold
Perceived creativity threat	High creativity threat	0.067	0.029	0.002	Very small; below practical necessity threshold
Perceived job insecurity	High job insecurity	0.061	0.021	0.002	Very small; below practical necessity threshold
Psychological safety	Low psychological safety	0.046	0.016	0.002	Very small; below practical necessity threshold
AI policy clarity	Low AI policy clarity	0.030	0.009	0.016	Negligible
AI/digital self-efficacy	Low AI/digital self-efficacy	0.030	0.006	0.002	Negligible
Trust in management	Low trust in management	0.078	0.036	0.002	Very small; below practical necessity threshold

Note. AI = artificial intelligence; NCA = Necessary Condition Analysis; CE-FDH = ceiling envelopment-free disposal hull; CR-FDH = ceiling regression-free disposal hull.

**Table 12:** Exploratory Bottleneck Table for AI Disclosure Silence.

Target Level of AI Disclosure Silence	AI Anxiety	Fear of Negative Evaluation	Perceived Creativity Threat	Perceived Job Insecurity	Low Trust in Management
50%	1.20	1.00	1.00	1.00	1.25
60%	1.20	1.40	1.60	1.00	1.25
70%	1.20	1.40	1.80	1.80	1.50
80%	2.20	1.40	1.80	2.20	1.75
90%	3.20	1.40	1.80	2.20	2.75

Note. AI = artificial intelligence.

**Table 13:** fsQCA Configurations Associated with High AI Disclosure Silence.

Condition	H1: Generalized Threat and Trust-Loss Pathway	H2: Evaluation-Insecurity and Weak-Guidance Pathway	H3: Creativity-Threat and Weak-Safety Pathway
AI anxiety	●	●	●
Fear of negative evaluation	●	●	●
Perceived creativity threat	●		●
Perceived job insecurity	●	●	
Low psychological safety		•	•
Low AI policy clarity		•	•
Low AI/digital self-efficacy			
Low trust in management	●	●	●
Consistency	0.963	0.960	0.940
Raw coverage	0.283	0.287	0.347
Unique coverage	0.026	0.030	0.090
Cases with strong membership	33	37	63

Note. AI = artificial intelligence; fsQCA = fuzzy-set Qualitative Comparative Analysis; ● = core presence; • = peripheral presence; blank = condition not relevant in the configuration. For protective variables, the calibrated low-condition forms were used. Therefore, low psychological safety, low AI policy clarity, low AI/digital self-efficacy, and low trust in management indicate weak protective conditions.

**Table 14:** fsQCA Configurations Associated with Low AI Disclosure Silence.

Condition	L1: Low-Threat and Trust Pathway	L2: Safe and Capable Disclosure Pathway	L3: Security and Support Pathway
AI anxiety	⊗	⊗	⊗
Fear of negative evaluation	⊗	⊗	⊗
Perceived creativity threat	⊗	⊙	
Perceived job insecurity	⊗		⊙
Low psychological safety		⊙	⊙
Low AI policy clarity	⊗	⊗	⊗
Low AI/digital self-efficacy		⊙	⊙
Low trust in management	⊗	⊗	⊗
Consistency	0.958	0.948	0.957
Raw coverage	0.338	0.304	0.307
Unique coverage	0.057	0.023	0.027

Note. AI = artificial intelligence; fsQCA = fuzzy-set Qualitative Comparative Analysis; ⊗ = core absence; ⊙ = peripheral absence; blank = condition not relevant in the configuration. Since low-condition forms were used for protective variables, the absence of low psychological safety indicates high psychological safety, the absence of low AI policy clarity indicates high AI policy clarity, the absence of low AI/digital self-efficacy indicates high AI/digital self-efficacy, and the absence of low trust in management indicates high trust in management.

**Table 15:** Machine-Learning Model Performance for Predicting AI Disclosure Silence.

Model	Dataset	R^2^	RMSE	MAE
Random Forest	Training set	0.847	0.598	0.474
Random Forest	Test set	0.414	1.137	0.884
XGBoost	Training set	0.744	0.773	0.609
XGBoost	Test set	0.427	1.125	0.854

Note. AI = artificial intelligence; R^2^ = coefficient of determination; RMSE = root mean square error; MAE = mean absolute error; XGBoost = extreme gradient boosting.

**Table 16:** SHAP-Based Predictor Importance for AI Disclosure Silence.

Rank	Predictor	Mean Absolute SHAP Value	Mean SHAP Value	Model-Based Direction
1	Fear of negative evaluation	0.354	0.029	Higher values increased predicted silence
2	AI anxiety	0.320	−0.091	
3	Perceived creativity threat	0.254	−0.017	
4	Trust in management	0.244	0.037	
5	AI policy clarity	0.161	−0.006	
6	Perceived job insecurity	0.149	−0.003	
7	Psychological safety	0.079	0.009	
8	AI/digital self-efficacy	0.046	−0.001	

Note. AI = artificial intelligence; SHAP = SHapley Additive exPlanations.

**Table 17:** Integrated Summary of Analytical Findings.

Condition	NCA Evidence	fsQCA Role	SHAP Relevance	Interpretation
AI anxiety	Below practical necessity threshold	Core in high-silence configurations	High predictive relevance	Important threat-related condition, but not indispensable alone
Fear of negative evaluation	Below practical necessity threshold	Core in high-silence configurations	Strongest predictive relevance	Central to the identity-protection and evaluation-risk logic
Perceived creativity threat	Below practical necessity threshold	Core in some high-silence pathways	High predictive relevance	Especially relevant in marketing because disclosure may threaten perceived originality
Perceived job insecurity	Below practical necessity threshold	Core in some high-silence pathways	Moderate predictive relevance	Relevant when AI disclosure is interpreted as a risk to employability or role value
Psychological safety	Below practical necessity threshold	Peripheral/protective in low-silence pathways	Lower but meaningful predictive relevance	Supports openness when combined with other positive conditions
AI policy clarity	Below practical necessity threshold	Protective in low-silence pathways	Moderate predictive relevance	Reduces uncertainty about acceptable AI use and disclosure expectations
AI/digital self-efficacy	Below practical necessity threshold	Protective in low-silence pathways	Lower predictive relevance	Helps employees feel capable of explaining and justifying AI-supported work
Trust in management	Below practical necessity threshold	Core in high- and low-silence configurations through its absence/presence	High predictive relevance	Key governance condition shaping whether disclosure feels safe and fairly interpreted

Note. AI = artificial intelligence; NCA = Necessary Condition Analysis; fsQCA = fuzzy-set Qualitative Comparative Analysis; SHAP = SHapley Additive exPlanations.

**Table 18:** Summary of Robustness, Sensitivity, and Validation Checks.

Check	Purpose	Result	Interpretation
Random Forest vs. XGBoost comparison	To examine whether predictive results depended on one model	XGBoost showed slightly stronger test-set performance than Random Forest	XGBoost was selected for SHAP-based interpretation
Five-fold cross-validation	To examine whether model performance depended on one train–test split	Both models showed similar cross-validated performance	Predictive results were reasonably stable
High vs. low fsQCA solutions	To examine configurational asymmetry	Low-silence configurations were not simple reversals of high-silence configurations	Supports the asymmetric and configurational logic of the study
NCA, fsQCA, and SHAP comparison	To examine consistency across different analytical logics	No condition reached the practical necessity threshold, but several conditions were important in fsQCA and SHAP	Supports the multi-pathway interpretation of AI disclosure silence
Measurement validation checks	To address construct overlap and the newly operationalized AI disclosure silence scale	EFA, CFA, CR, AVE, and HTMT supported construct distinction	Provides initial support for AI disclosure silence as a separate construct

Note. AI = artificial intelligence; NCA = Necessary Condition Analysis; fsQCA = fuzzy-set Qualitative Comparative Analysis; SHAP = SHapley Additive exPlanations; EFA = exploratory factor analysis; CFA = confirmatory factor analysis; AVE = average variance extracted; HTMT = heterotrait–monotrait ratio; RMSE = root mean square error; MAE = mean absolute error; R^2^ = coefficient of determination.

## Data Availability

The original contributions presented in this study are included in the article/Further inquiries can be directed to the corresponding author.
